# Investigation of the Nature of CgCDPK and CgbHLH001 Interaction and the Function of bHLH Transcription Factor in Stress Tolerance in *Chenopodium glaucum*

**DOI:** 10.3389/fpls.2020.603298

**Published:** 2021-01-22

**Authors:** Zixin Zhou, Juan Wang, Shiyue Zhang, Qinghui Yu, Haiyan Lan

**Affiliations:** ^1^Xinjiang Key Laboratory of Biological Resources and Genetic Engineering, College of Life Science and Technology, Xinjiang University, Urumqi, China; ^2^Institute of Horticulture Crops, Xinjiang Academy of Agricultural Sciences, Urumqi, China

**Keywords:** bHLH function, CgCDPK and CgbHLH001 interaction, *Chenopodium glaucum*, phosphorylation, stress tolerance

## Abstract

Calcium-dependent protein kinase (CDPK) and its substrates play important roles in plant response to stress. So far, the documentation on the characterization of the CDPK and downstream interaction components (especially transcription factors, TFs) is limited. In the present study, an interaction between CgCDPK (protein kinase) (accession no. MW26306) and CgbHLH001 (TF) (accession no. MT797813) from a halophyte *Chenopodium glaucum* was further dissected. Firstly, we revealed that the probable nature between the CgCDPK and CgbHLH001 interaction was the phosphorylation, and the N-terminus of CgbHLH001, especially the 96th serine (the potential phosphorylation site) within it, was essential for the interaction, whereas the mutation of ^96^Ser to alanine did not change its nuclear localization, which was determined by the N-terminus and bHLH domain together. Furthermore, we verified the function of *CgbHLH001* gene in response to stress by ectopic overexpression in tobacco; the transgenic lines presented enhanced stress tolerance probably by improving physiological performance and stress-related gene expression. In conclusion, we characterized the biological significance of the interaction between CDPK and bHLH in *C. glaucum* and verified the positive function of CgbHLH001 in stress tolerance, which may supply more evidence in better understanding of the CDPK signaling pathway in response to adversity.

## Introduction

Calcium-dependent protein kinase (CDPK) converts calcium signal into the physiological responses by phosphorylating various substrates including ion channel proteins, transcription factors, and metabolic enzymes (Yip Delormel and Boudsocq, 2019). The large diversity of targets confers pivotal roles of CDPKs in the regulation of plant growth, development, and tolerance to stresses (Li M. Y. et al., 2020). The transient change of the cytosolic Ca^2+^ level can be sensed by diverse CDPKs, which consequently activates the specific endogenous substrates by phosphorylation cascades (Harper et al., 2004; Schulz et al., 2013; Ranty et al., 2016; Ormancey et al., 2017). CDPKs consist of at least four conserved modules as: a variable N-terminal domain (VNTD), a serine/threonine protein kinase domain, an autoinhibitory junction domain (JD) and the C-terminal calmodulin-like domain (CaMLD) composed of four EF-hand Ca^2+^-binding motifs (Harper et al., 2004). *In vivo* activation of CDPKs is driven by conformational change induced by Ca^2+^ binding, which results in the release of pseudo-substrate from the active site of the kinase domain (Harmon et al., 1994; Harper et al., 1994). The VNTD plays an important role in substrate recognition, which is indispensable for NtCDPK1 in interaction and subsequent phosphorylation of RSG (repression of shoot growth) transcription factor (Ito et al., 2010). CDPKs combine both Ca^2+^ sensing by EF-hand calcium-binding motifs and activating by the protein kinase domain, and directly transmit Ca^2+^ signals into phosphorylation events (Yip Delormel and Boudsocq, 2019).

Accumulated evidence has been documented on the interaction between CDPKs and its downstream substrates (e.g., transcription factors) in response to abiotic stress (Rodriguez Milla et al., 2006; Uno et al., 2009; Mehlmer et al., 2010). Further characterization of the specific targets of CDPK and the biological significance in their interaction (e.g., phosphorylation) are much important for understanding the molecular mechanism of the CDPK signaling pathway (Boudsocq and Sheen, 2013; Yip Delormel and Boudsocq, 2019). Phosphorylation is a common way in the posttranslational modification and regulation of transcription factors (Ubersax and Ferrell, 2007). NtCDPK1 in tobacco has been reported with phosphorylation of a basic leuzipper TF—RSG by which the 14–3–3 proteins are bound (Nakata et al., 2009), such an interaction negatively regulates RSG to sequester in the cytoplasm in response to GAs. Inhibition of *NtCDPK1* expression represses the GA-induced phosphorylation at the 114th serine of RSG and its translocation from nucleus to cytoplasm (Igarashi et al., 2001; Ishida et al., 2008). OsCDPK14 has been identified to interact with and phosphorylate OsDi19-4 (drought-induced 19) in rice, which consequently improves the function of OsDi19-4 in regulating downstream ABA-responsive genes (Wang et al., 2016). In consistence with that in rice, AtCPK11 is also shown in phosphorylating AtDi19-1 in Arabidopsis (Liu et al., 2013), and overexpression of *AtDi19-1* promotes drought tolerance while the *di19-1* mutant is hypersensitive to drought. So far, no report has been documented in the interaction between CDPK and bHLH TF and the phosphorylation effect.

The bHLH superfamily is the second largest class of plant TFs (Feller et al., 2011), which contains two conserved and functionally distinct regions: the N-terminal basic region and the middle HLH region (Atchley and Fitch, 1997). The former functions in recognizing and specifically binding the DNA motif on the target gene promoter (Atchley et al., 1999), while the HLH region provides sequence-specific DNA recognition and mediates domain dimerization (Nair and Burley, 2000). A large group of plant bHLH TFs contain a basic region allowing them to recognize the E-Box (5′-CANNTG-3′) and/or G-Box (5′-CACGTG-3′) motifs and recruit coactivator or corepressor complexes to regulate the downstream gene expression (Carretero-Paulet et al., 2010; Yang J. et al., 2020). bHLH TFs have been proved to be involved in a variety of regulatory processes, including plant growth and development, metabolism regulation, and signal transduction, and in response to stresses (Feng et al., 2012; Huang et al., 2013; Zhai et al., 2016). For example, *ICE1* (inducer of CBF expression) and *ICE2* in *Arabidopsis thaliana*, *PubHLH1* in *Pyrus ussuriensis*, and *MdCIbHLH1* in *Malus domestica* have been reported in response to cold stress (Chinnusamy et al., 2003; Feng et al., 2012; Kurbidaeva et al., 2014; Jin et al., 2016); *PtrbHLH* in *Poncirus trifoliata* modulates peroxidase-mediated scavenging of hydrogen peroxide (Huang et al., 2013); *OsbHLH148* in *Oryza sativa* functions in drought tolerance as a component of the jasmonate signaling module (Seo et al., 2011). Besides, the bHLH genes (*bHLH38*, *bHLH39*, *bHLH100*, *bHLH101*) in Arabidopsis are reported working in iron acquisition and heavy metal detoxification (Yuan et al., 2008; Sivitz et al., 2012; Wu et al., 2012). To date, plant bHLH TFs have been associated with various abiotic stresses by participating in the regulation of gene expression (Castilhos et al., 2014). However, the regulatory mechanisms of bHLH TFs in halophytes remain unclear.

In the previous work, we preliminarily identified the interaction between CgCDPK and CgbHLH001 in *Chenopodium glaucum* (Wang et al., 2017), an annual halophyte distributed in semi-arid and saline areas in Xinjiang, China (Institute of Botany, Academia Sinica, 1983). To further explore the biological meaning of CgCDPK and CgbHLH001 interaction, and the function of CgbHLH001 in response to abiotic stress, in the present study, we tried to address the following questions: (1) What is the nature of the interaction between CgCDPK and CgbHLH001? (2) What specific domain of CgbHLH001 is responsible for the interaction with CgCDPK? (3) What functions does CgbHLH001 have in response to stress? Figuring out these questions may help us understand the biological significance of interaction between CgCDPK and CgbHLH001 and the function of CgbHLH001 in stress regulation.

## Materials and Methods

### Plant Cultivation and Treatments

Mature seeds of *Nicotiana tabacum* were surface-sterilized and then submerged in sterilized H_2_O and left at 4°C in a refrigerator for 3 days in the dark for uniform germination. For seed germination under NaCl and PEG stresses, the seeds were sown on MS medium containing different concentrations of NaCl—0, 50, 100, 200 mmol⋅L^–1^, or PEG 6,000—0, 5, 10, 15%, and placed at 25°C, 30–40% relative humidity (RH), in a 16-h light/8-h dark photoperiod, and 100 μmol⋅m^–2^ s^–1^ illumination for 14 days, and the germination percentage and seedling growth were recorded and calculated. The seedlings were then transferred into pots containing a 3:1 mixture of vermiculite: perlite (V/V) under conditions of 25–28°C, 20–30% RH, 16 h light/8 h dark photoperiod, and 170–180 μmol⋅m^–2^ s^–1^ illumination, and cultivated for 4–6 weeks with well-watering and supplying with Hoagland solution at an interval of 2–3 weeks. For plants used in “Bimolecular fluorescence complementation assay” (BiFC) and “Luciferase complementation imaging assay” (LCI), *N. tabacum* was replaced with *N. benthamiana*; the cultivation manipulation was similar to the above procedure.

For phenotypic analysis of drought stress, 3–4-week-old plants (early described) of *N. tabacum* T2 transgenic lines of *CgbHLH001* overexpression (OE1, OE2, OE3, OE5) and non-transgenic plants (NC89, NT) were subjected to natural drought conditions without watering under similar conditions as above till showing significant wilting symptoms (about 3–4 weeks); the plants were then recovered by fully watering for 1 week, and the survived leaves and dry-withered leaves of each OE and all NT plants were separately recorded. The normally watered plants were used as control. The correlation between transgenic lines and survival leaves was analyzed by Chi-square (*χ*^2^)-test.

For qRT-PCR analysis of *NtCDPK* expression in *CgbHLH001*-overexpressed lines, 6 week-old plants (early described) of *N. tabacum* T2 transgenic lines (OE2, OE3, OE5), and NT were applied with 300 mmol⋅L^–1^ NaCl, 15% PEG 6,000, or 4°C treatment and sampled at 12 h with the upper part young leaves; three biological replicates were applied for each treatment; and the samples were immediately frozen in liquid nitrogen and stored at −80°C until further use.

For ROS staining, seedlings of *N. tabacum* (NC89) grown on MS medium for 2 weeks (early described) were used for DAB (3,3′-diaminobenzidine) or NBT (nitro blue tetrazolium) staining. 10 uniform seedlings were placed in 50 mL MS solution added with 20% PEG 6,000 or 200 mmol⋅L^–1^ NaCl and shaken at 100 rpm in a light incubator for 5 h, followed by submergence in DAB and NBT staining solution.

For physiological assay and gene expression analysis in response to stresses, 6 week-old plants (early described) of *N. tabacum* T2 transgenic lines of *CgbHLH001* overexpression (OE1, OE2, OE3, OE5) and NT were used in measurement. For NaCl or PEG 6,000 treatments, 200 mmol⋅L^–1^ NaCl or 20% PEG 6,000 in half-strength Hoagland solution was applied to the pot plants avoiding spilling on the leaf; after being saturated with the solution, the plants were remained in the tray with treatment solution for 24 h and then sampled. For the 4°C treatment, the above plants were placed in a plant light incubator at 4°C for 24 h and then sampled. The half-strength Hoagland solution-treated plants were used as control. Three biological replicates were applied to all treatments. All samples were immediately frozen at −80°C for further use.

### Yeast Two-Hybrid (Y2H) Analysis

cDNA sequences of *CgCDPK* and *CgbHLH001* were inserted into pBT3-N and pPR3-N, respectively, and constructed pBT3-*CgCDPK* and pPR3-*CgbHLH001* yeast expression vectors. The mixture of both vectors was transformed into the yeast strain NMY51 competent cells which (200 μL) were then spread on the yeast synthetic dropout medium without Leu and Trp (SD/-Trp-Leu) and incubated at 30°C for 2–4 days to identify the transformants. Three single clones from SD/-Trp-Leu plates were transferred to SD/-Trp-Leu-His-Ade plates (SD medium without tryptophan, leucine, histidine, and adenosine). A single clone was incubated in SD/-Trp-Leu broth overnight; 5 mL of the cultures were then removed and centrifuged, and the pellet was washed and suspended with distilled water, and the suspensions (diluted into OD_600_ values as 1.0, 0.1, and 0.01, respectively) were dropped onto plates for further growth. To test the activation of the reporter gene β-*galactosidase*, a sterilized filter paper was properly covered on the above medium and carefully pressed with the glass spreader to drive out the bubbles and make full contact in between; after marking the colony position, the filter paper was then taken out carefully with forceps and immediately placed into liquid nitrogen for 10 s and then taken out to thaw, repeated freezing, and thawing steps again, then the contact side of the filter paper was faced up and pressed against another filter paper rinsed with 2 mL of Z buffer + X-gal, and the excess buffer was removed. The two layers of filter paper were placed at 28°C in an incubator to develop the color.

### Determination of the Localization of CgbHLH001

The localization was predicted by cNLS Mapper^1^. Different truncated fragments of *CgbHLH001* and full length of Δ*CgbHLH001*(*^96^S-A*) cDNA were constructed into the pSuper1300*-MCS-GFP* plant expression vector, which were then transformed into *Agrobacterium tumefaciens* strain GV3101 by electroporation. The recombinant *A. tumefaciens* strains containing different constructs were cultivated, harvested, and resuspended in infiltration buffer (10 mmol⋅L^–1^ MES, 0.2 mmol⋅L^–1^ acetosyringone, and 10 mmol⋅L^–1^ MgCl_2_) at a final OD_600_ value of 0.8. The pSuper1300*-cDNAs-GFP*/GV3101 [cDNAs represent nucleotide sequences of 1–146 aa, 1–197 aa, 147–263 aa, 198–263 aa, full length of CgbHLH001, or ΔCgbHLH001(^96^S-A)] (A) were thoroughly mixed with pSuper1300*-CBL-RFP*/GV3101 (CBL: calcineurin B-like protein, membrane marker; RFP: red fluorescent protein) (B) and pSuper1300*-P19*/GV3101 (to promote protein expression) (C) according to the volume proportion as 450 μL (A): 450 μL (B): 300 μL (C) and then left at 28°C for 2 h in the dark. Five- to six-week-old *N. benthamiana* plants (early described) were used in infiltration. The mixture of different *Agrobacterium* strains was infiltrated into the fresh leaves (Wang et al., 2017), and the infiltration areas were labeled for recognition. Treated plants were left in the dark overnight and then transferred to the normal growth conditions for 72 h. The fluorescent signals in the epidermal cells of *N. benthamiana* leaves were inspected under the confocal microscope (Zeiss LSM 800, Jena, Germany) at 517 nm (for GFP) and 572 nm (for RFP), respectively. For testing the localization under different stresses, the abscised leaves were suspended on the surface of 200 mmol⋅L^–1^ NaCl, 10 μmol⋅L^–1^ ABA, 20% PEG 6,000 solution, or ddH_2_O and incubated for 30 min or 6 h at room temperature. Three replicates were applied to each treatment.

### Luciferase Complementation Imaging (LCI) Assay

cDNA sequences of *CgCDPK* and *CgbHLH001* were constructed into pCAMBIA-nLUC and pCAMBIA-cLUC (nLUC and cLUC: N and C termini of luciferase) plant expression vectors, respectively, which were then transformed into *A. tumefaciens* strain GV3101 by electroporation. The recombinant *A. tumefaciens* strains were cultivated, harvested, and resuspended in infiltration buffer (10 mmol⋅L^–1^ MES, 0.2 mmol⋅L^–1^ acetosyringone, and 10 mmol⋅L^–1^ MgCl_2_) at a final OD_600_ value of 0.8. *N. benthamiana* plant (5–6-week-old, early described) leaves were infiltrated with the combinations of different *A. tumefaciens* recombinant strains harboring with n-LUC + c-LUC, c-LUC + CDPK-n-LUC, n-LUC + bHLH-c-LUC, CDPK-n-LUC + bHLH-c-LUC (leaf was equally divided into four quadrants and labeled). Three days later, treated leaves were sprayed with luciferase substrate-luciferin in the dark for 5 min to quench the fluorescence and then placed in a low-light cooled CCD imaging apparatus (Lumazone 1300B, Princeton Instruments, United States) to capture the LUC images.

### Bimolecular Fluorescence Complementation (BiFC) Assay

cDNA sequences of *CgCDPK* and *CgbHLH001* were constructed into pSPY-NE and pSPY-CE plant expression vectors, respectively; the different truncated fragments of *CgbHLH001* and the full length of Δ*CgbHLH001*(*^96^S-A*) cDNA were inserted into pSPY-CE, which were then transformed into *A. tumefaciens* strain GV3101. The pSPY*-CgCDPK-NE/*GV3101 (A) was thoroughly mixed with pSPY-*cDNAs-CE/*GV3101 [cDNAs represent nucleotide sequences of 1–146 aa, 1–197 aa, 147–263 aa, 198–263 aa, the full length of CgbHLH001 and ΔCgbHLH001(^96^S-A)] (B), pSuper1300*Pro:CBL-RFP*/GV3101 (C), and pSuper1300*Pro:P19*/GV3101 (D) according to the volume proportion as: 450 μL (A): 450 μL (B): 450 μL (C): 300 μL (D) and then kept at 28°C for 2 h. The following procedures were the same as “Determination of the localization of CgbHLH001.” Three replicates were applied to each treatment.

### Phosphorylation Measurement *in vitro*

Expression and purification of CgCDPK and CgbHLH001 proteins were performed according to the method as described previously (Wang et al., 2017). Purified CgCDPK (1 μg) and CgbHLH001 (1 μg) were mixed with 6 μL reaction buffer [5 × phosphorylation reaction buffer: 125 mmol⋅L^–1^ Tris–HCl (pH 7.5), 50 mmol⋅L^–1^ MgCl_2_, 5 mmol⋅L^–1^ CaCl_2_, 5 mmol⋅L^–1^ DTT] to a total volume of 30 μL, and then the ^32^P-labeled ATP was added to a final concentration of 2.5 μmol⋅L^–1^ (5 μCi γ-^32^P-ATP); after mixing gently, the mixture was first incubated at 25°C to react for 10 min and at 30°C for 20 min, and then 5 μL of 6 × SDS loading buffer was added and the proteins (in the mixture) were fully denatured at 95°C for 5 min, then cooled down on ice. The phosphorylation reaction mixture (20 μL) was subjected to SDS-PAGE under 120 V for 2 h. The gel was rinsed with ddH_2_O for 3 times under gentle shaking to remove the radioisotopes; after staining by Coomassie brilliant blue and destaining, the gel was photographed and scanned by a multifunctional laser imager (Typhoon 9410, Amersham, United States) to detect autoradiography with a phosphor screen.

### Site-Directed Mutagenesis Analysis

The phosphorylation sites of CgbHLH001 were predicted by using the Kinase Phos 2.0 program^2^. The most probable phosphorylation site for CgCDPK to phosphorylate CgbHLH001 was predicted at ^91^GKRLKS^96^, and the 96th serine was considered as the amino acid (aa) to be phosphorylated. To verify the importance of this aa, we changed the codon TCA (Serine) to GCC (Alanine). The primers were designed based on the changed nucleotides (Supplementary Table 1); the target gene *CgbHLH001* was ligated into the intermediate vector pGEM-T-easy, according to the instructions of gene site-directed mutagenesis kit (Cat. CL302, Biomed, Beijing, China), in a total volume of 50 μL containing 10 μL of 5 × Xerox DNA polymerase buffer, 1 U Xerox DNA polymerase, 1 μL 10 mmol⋅L^–1^ dNTP mixture, plasmid template, and the primers, which were mixed well to perform PCR analysis. The finished reaction products were then added with site-directed mutagenesis enzyme (1 μL) and incubated at 37°C for 3 h to degrade the non-mutated templates. The site-directed mutated plasmid (10 μL) was transformed into *E. coli* competent cells XL10-Gold; after being identified by PCR, the plasmid was sequenced. The confirmed changed sequence was constructed into the corresponding plant expression vectors.

### Quantitative RT-PCR Analysis of Gene Expression

The total RNA was isolated from the seedlings or young leaves using Plant RNA kit (Omega, United States) according to the manufacturer’s instructions. Approximately 1 μg of total RNA was reversely transcribed into cDNA using the TransScript All-in-One First-Strand cDNA Synthesis SuperMix for qPCR (TransGen, Beijing, China) according to the manufacturer’s instructions. qRT-PCR was used to measure transcript levels of *CgbHLH001* and stress-related genes. The primers were shown in Supplementary Table 1. qRT-PCR analysis was performed in a LightCycler 96 Real-Time System (Roche, United States); the PCR reaction conditions were as follows: 94°C 30 s; 40 cycles of 94°C 5 s, 60°C 30 s. qPCR was performed with PerfectStart Green qPCR SuperMix kit (TransGen, Beijing, China). Three biological replicates with two technical replicates of each were applied to each treatment, and the 2^–ΔΔCT^ method (Shi and Chiang, 2005) was employed to calculate the relative expression level of each gene. *Ntactin* was used as an internal reference for tobacco to normalize the expression level. The relative quantification was described as fold change of gene expression in the test sample compared to the control.

### Generation of *CgbHLH001*-Overexpressed Transgenic Tobacco Lines

*CgbHLH001* cDNA was constructed into the plant expression vector pCAMBIA2300 driven by the *CaMV35S* promoter, which was then transformed into the *A. tumefaciens* strain EHA105 and used for transformation of leaf disks of *N. tabacum* (Horsch et al., 1985). The kanamycin-resistant plantlets (T0 generation) regenerated from the explants were identified by PCR and RT-PCR; seed germination under stress treatments was performed with T1 generation in accordance with segregation ratio 1:3; four *CgbHLH001*-overexpressed lines—OE1, OE2, OE3, OE5 T2 generation—were obtained, which were then used in various experiments in the present study. The primers used in identification were present in Supplementary Table 1.

### Measurements of Physiological Parameters

#### *In situ* Accumulation of H_2_O_2_ and Superoxide Anion

Histochemical staining by DAB (3,3′-diaminobenzidine) for H_2_O_2_ or NBT (nitro blue tetrazolium) for superoxide anion was employed in the measurements. Two-week-old plants of transgenic and non-transgenic tobacco (early described) were soaked in aqueous MS medium with 200 mmol⋅L^–1^ NaCl or 20% PEG 6,000 for 5 h before staining; the MS only was used as control. Plants were incubated in 0.1% DAB solution (0.1 g DAB in 100 mL ddH_2_O, adjusted *pH*-value to 3.8 with HCl) or 0.2% NBT solution (0.1 g NBT in 50 mL 50 mmol⋅L^–1^ sodium phosphate buffer, pH 7.5) for overnight at room temperature in the dark according to the method described by Kumar and Sinha (2013). Then the plants stained by DAB or NBT were immersed into 75% ethanol in a boiling water bath (with carefully shaking from time to time) till the leaves were cleared of chlorophyll. Ten plants from each treatment were stained and the photographs were taken.

#### Reactive Oxygen Species Level and Lipid Peroxidation

For detection of superoxide (⋅O_2_^–^), hydrogen peroxide (H_2_O_2_), and malondialdehyde (MDA) accumulation, young fresh leaves (0.15 g) were homogenized in ice-cold normal saline to form 10% homogenates and measured according to the manufacturer’s protocols of the assay kits [Cat. A052 (⋅O_2_^–^); A064 (H_2_O_2_); A003 (MDA); Nanjing Jiancheng Bioengineering Institute, Nanjing, China]; the absorbance values of ⋅O_2_^–^, H_2_O_2_, and MDA were measured at 550, 405, and 532 nm, respectively. The ⋅O_2_^–^ production rate, H_2_O_2_ concentration, or amount of MDA was calculated and expressed as μmol⋅mg^–1^ protein.

#### Osmolyte Concentration

For determination of proline, soluble sugar (SS) or glycinebetaine (GB) concentration, young fresh leaves (0.2 g) were sampled and immediately dried at 80°C for 5 h and then ground into homogeneous powder for further determination. Proline and SS concentrations were determined by spectrophotometry using assay kits [Cat. A107 (proline); A145 (SS); Nanjing Jiancheng Bioengineering Institute, Nanjing, China]; GB was determined by spectrophotometry according to the method described by Lokhande et al. (2010). The absorbance values of proline, SS, and GB were measured at 515, 630, and 525 nm, respectively.

#### Activity of Antioxidant Enzymes and Concentration of Non-enzymatic Antioxidants

Young fresh leaves (0.2 g) were ground into homogenates and suspended in extraction buffer, which was then centrifuged at 4°C for 10 min, and the supernatant was immediately used for the determination of the activity of antioxidant enzymes [superoxide dismutase (SOD), peroxidase (POD), catalase (CAT), ascorbate peroxidase (APX), glutathione reductase (GR)] and the content of non-enzymatic antioxidants [reduced glutathione (GSH) and ascorbic acid (AsA)] using the assay kits [Cat. A001 (SOD); A084 (POD); A007 (CAT); A062 (GR); A123 (APX); A006 (GSH); A009 (AsA); Nanjing Jiancheng Bioengineering Institute, Nanjing, China]. The enzyme activity was expressed as unit⋅mg^–1^ protein, and the antioxidant content was expressed as μg⋅mg^–1^ protein.

#### Stomatal Closure Assay

Plants (5–6-week-old, early described) of *N. tabacum* T2 transgenic lines of *CgbHLH001* (OE1, OE2, OE3, OE5) and NT were subjected to the stomatal aperture measurement as described previously (Zou et al., 2015). Fully expanded young leaves were floated in the stomatal closure solution (20 mmol⋅L^–1^ KCl, 1 mmol⋅L^–1^ CaCl_2_, 5 mmol⋅L^–1^ MES-KOH, pH 6.15) and kept under light (25°C, 450 μmol⋅m^–2^ s^–1^) for 2.5 h, followed by addition of 10 μmol⋅L^–1^ ABA or ddH_2_O to the above solution for another 2.5 h. Then, the abaxial epidermal layers were peeled quickly to make slides, and the stoma were inspected and photographed with a Leica microscope (Leica DFC320, Germany). Stomatal aperture was measured by ImageJ software (National Institutes of Health).

#### Water Loss

Plants (4-week-old, early described) of *N. tabacum* T2 transgenic lines of *CgbHLH001* (OE1, OE2, OE3, OE5) and NT were used in water loss measurement. The fresh leaves were detached from the plants in similar positions and weighed immediately, then placed on a piece of foil paper and kept at 25°C, 20–30% RH, without disturbance. The leaves were weighed at a regular interval of 30 min for a total of 300 min. Four replicates (four leaves) of each OE line or NT plant were employed in the experiment.

### Thermal Imaging

Plants (3-week-old, early described) of *N. tabacum* T2 transgenic lines of *CgbHLH001* (OE1, OE2, OE3, OE5) and NT were used for thermal imaging according to the method previously described (Zou et al., 2015). Plants were subjected to natural drought (without watering) under normal conditions for 1 week, the images were acquired by using the infrared thermal imager (VarioCAM HD, Germany), and leaf temperature was calculated by IRBIS 3 software.

### Statistical Analysis

All data were analyzed using Microsoft Excel 2016 and the software of GraphPad Prism 7.0 (GraphPad Software, San Diego, United States). One-way and two-way ANOVA were used to test the significance of main effects. Differences were compared by Tukey multiple comparison test at 0.05, 0.01, or 0.001 significance level.

## Results

### The Probable Nature of the Interaction Between CgCDPK and CgbHLH001

**FIGURE 1:**
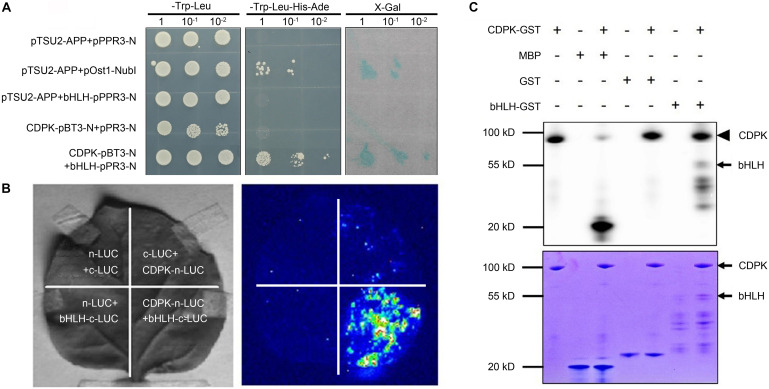
Validation of the interaction and the probable nature between CgCDPK and CgbHLH001. **(A)** Identification of the interaction by Y2H assay. pTSU2-APP/pPR3-N, pTSU2-APP/bHLH-pPR3-N, CgCDPK-pBT3-N/pPR3-N: negative control; pTSU2-APP/pOst1-NubI: positive control; CDPK-pBT3-N + bHLH-pPR3-N: test group. 1, 10^–1^, 10^–2^: yeast culture dilution for 0, 10, 100 folds. **(B)** *In vivo* interaction detection by LCI assay. Left: leaves infiltrated with different agrobacterial combinations; right: LUC images corresponding to the “Left” leaves. n-LUC + bHLH-c-LUC, CDPK-n-LUC + c-LUC, n-LUC + c-LUC: negative controls; CDPK-n-LUC + bHLH-c-LUC: the test combination. **(C)** *In vitro* phosphorylation analysis. +, – on the top panel: represent the presence or absence of the components on the left; upper panel: phosphor screen result, the solid arrowhead points to the autophosphorylation band of CgCDPK, the black arrow points to the phosphorylated bands of CgbHLH001; lower panel: Coomassie brilliant blue staining, the black arrows point to the positions of CgCDPK-GST and CgbHLH001-GST proteins.

In the previous work, we preliminarily identified the interaction between CgCDPK and CgbHLH001 in *C. glaucum* by pulldown and BiFC (Wang et al., 2017); in the present study, further verification of the specific interaction was performed by Y2H analysis and LCI assay to explore the nature of CgCDPK and CgbHLH001 interaction (Figures 1A,B). Our results showed that in Y2H, pBT3-N-*CgCDPK* (bait) combined with pPR3-N-*CgbHLH001* (prey) could grow on SD/-Trp-Leu-His-Ade medium, and presented apparently β-galactosidase activity, which means that these proteins can interact in yeast by activating the expression of the reporter gene. Meanwhile, LCI assay also revealed a striking interaction between CgCDPK and CgbHLH001 *in vivo*. Our results in the present study combined with the previous data confirmed the existence of the interaction between CgCDPK and CgbHLH001. As a protein kinase, CDPK plays an important role in plant signal transduction by phosphorylating the substrate (often TF) (Nakata et al., 2009; Wang et al., 2016). To clarify the nature of the interaction between CgCDPK (kinase) and CgbHLH001 (TF), we performed the *in vitro* phosphorylation assay with the purified proteins of CgCDPK-GST and CgbHLH001-GST. Results showed that co-incubation of CgCDPK and CgbHLH001 could induce CgCDPK itself and CgbHLH001 strongly phosphorylated (Figure 1C).

### Effects of CgbHLH001 Domains on Localization and Interaction With CgCDPK

#### Changes of the Subcellular Localization of Truncated CgbHLH001

**FIGURE 2:**
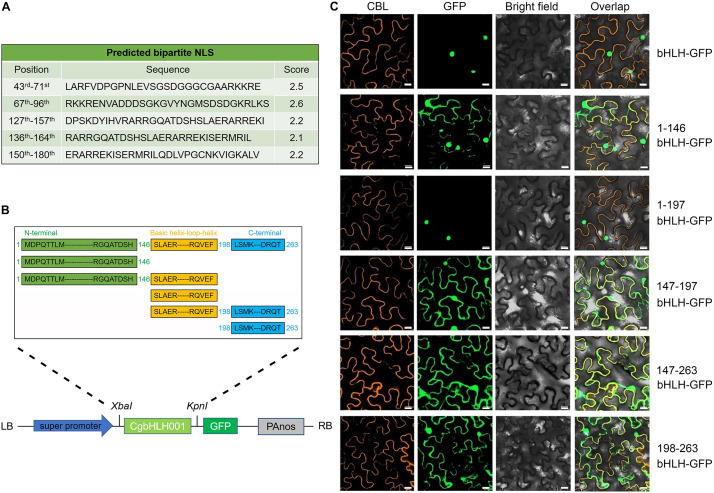
Subcellular localization of different domains of CgbHLH001. **(A)** Prediction of NLS sequences of CgbHLH001. **(B)** Schematic diagram of different deletions of CgbHLH001 domains and the construction of the plant expression vector for subcellular localization. The green, orange, and blue regions represent N terminal, basic helix–loop–helix, and C terminal domains, respectively. **(C)** BiFC assay testing the localization. CBL: calcineurin B-like protein, membrane marker; GFP: green fluorescence protein; bright field: visible light; overlap: merged bright field with CBL and GFP. bHLH-GFP: full length of CgbHLH001 fused to GFP; 1–146 bHLH: 146 aa of CgbHLH001 N terminus; 1–197 bHLH: 197 aa of N terminus + bHLH domain of CgbHLH001; 147–197 bHLH: 147–197 aa bHLH domain of CgbHLH001; 147–263 bHLH: 147–263 aa bHLH domain + C terminus of CgbHLH001; 198–263 bHLH: 198–263 aa C terminus of CgbHLH001. Bar = 20 μm.

In the previous work, CgbHLH001 was confirmed with the nuclear localization (Wang et al., 2017). By further analysis of the nuclear localization signal (NLS), we found that the bipartite NLSs of CgbHLH001 were distributed at 43rd–180th aa (263 aa in total) within the N terminus and the basic helix–loop–helix domain (Figure 2A). To clarify the function of different domains, we made a series of deletion to the amino acid sequence of CgbHLH001 [full length (1–263 aa), C terminal deletion [1–146 aa (N terminus), and 1–197 aa (N terminus + bHLH domain)], N and C terminal deletion [147–196 aa (bHLH domain)], and N terminal deletion [147–263 aa (bHLH + C terminus) and 198–263 aa (C terminus)] and then determined the subcellular localization (Figure 2B). The observation of the fluorescence signal showed that the full length and 1–197 aa of CgbHLH001 was located in the nucleus exclusively, while other fragments were found in the whole cytoplasm and nucleus (Figure 2C), our results suggest that the N terminus and the bHLH domain together are essential for the nuclear localization of CgbHLH001, which is also consistent with the distribution of the NLSs at N terminal and bHLH domains (Figure 2A).

**FIGURE 3:**
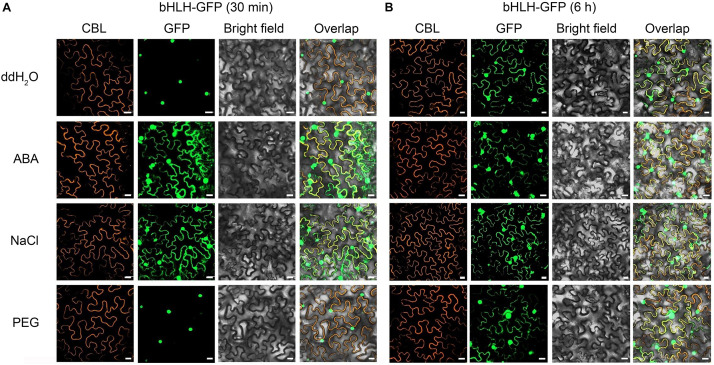
Effects of different abiotic stresses on subcellular localization of CgbHLH001. **(A)** Effect of short time (30 min) treatment. **(B)** Effect of longer time (6 h) treatment. CBL: calcineurin B-like protein, membrane marker; GFP: green fluorescent protein; bright field: visible light; overlap: merged bright field with CBL and GFP. bHLH-GFP, full length of CgbHLH001 fused to GFP. Bar = 20 μm.

To investigate the effect of exogenous stimulus on localization of CgbHLH001, *N. benthamiana* leaves were exposed to abiotic stress after co-expressed with CgbHLH001-GFP + CBL + P19. As shown in Figure 3A, the GFP fluorescence of untreated CgbHLH001 was found in the nucleus, while the green signal was observed in the whole cytoplasm and nucleus when treated with 10 μmol⋅L^–1^ ABA or 200 mmol⋅L^–1^ NaCl for 30 min (no effect with ddH_2_O or 20% PEG 6,000 treatment). When extending the treatment time to 6 h, all these treatments made CgbHLH001 present in the cytoplasm and nucleus (Figure 3B), which suggests that CgbHLH001 can respond to stress.

#### Analysis of the Interaction Between CgCDPK and Different Domains of CgbHLH001

**FIGURE 4:**
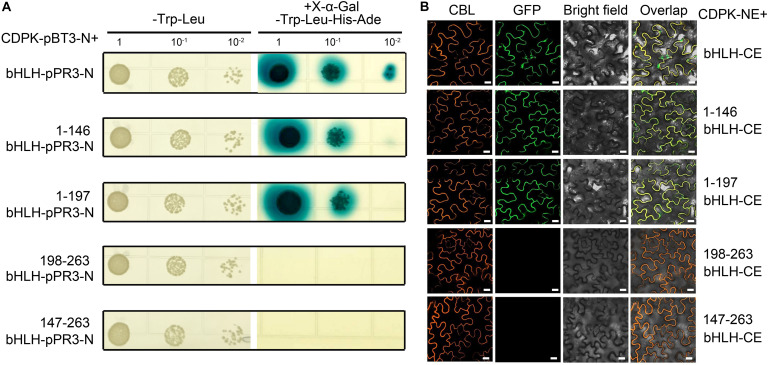
Verification of interaction between CgCDPK and different truncated domains of CgbHLH001. **(A)** Interaction test by Y2H assay. CDPK-pBT3-N + bHLH-pPR3-N, 1–146 bHLH-pPR3-N, 1–198 bHLH-pPR3-N, 198–263 bHLH-pPR3-N, 147–263 bHLH-pPR3-N: *CgCDPK* in yeast expression vector pBT3-N and full length or different truncated fragments of *CgbHLH001* in the pPR3-N vector. 1, 10^–1^, 10^–2^: yeast culture dilution for 1-, 10-, 100-fold. **(B)** BiFC assay. CDPK-NE, CgCDPK fused with N terminus of GFP; bHLH-CE, CgbHLH001 fused with C terminus of GFP. 1–146 bHLH-CE, 1–197 bHLH-CE, 147–263 bHLH-CE, and 198–263 bHLH-CE: different truncated fragments of CgbHLH001 fused with C terminus of GFP. CBL: calcineurin B-like protein, membrane marker; GFP: green fluorescence protein; bright field: visible light; overlap: merged bright field with CBL and GFP. Bar = 20 μm.

To determine the key domain of CgbHLH001 functioned in the interaction, the different truncated fragments of CgbHLH001 were tested of the correlation with CgCDPK (as a bait) in Y2H assay (Figure 4); results showed that 1–146 (aa) N terminus or 1–197 (aa) (N terminus + bHLH domain) of CgbHLH001 could interact with CgCDPK by growing on selective SD medium/-Trp-Leu-His-Ade and forming the blue colony (Figure 4A, the 2nd and 3rd rows), and it was confirmed by BiFC assay with the presence of the fluorescence signal between CgCDPK and these two truncated domains of CgbHLH001 (Figure 4B, the 2nd and 3rd rows). However, no interaction was observed between CgCDPK and the other truncated domains of CgbHLH001 in both Y2H or BiFC test (Figures 4A,B, the 4th and 5th rows). Taken together, these results suggest that the N terminal domain is essential for the interaction between CgCDPK and CgbHLH001.

#### Effect of the Amino Acid Sequence Mutation of the N Terminal Domain on Subcellular Localization of CgbHLH001 and Interaction With CgCDPK

**FIGURE 5:**
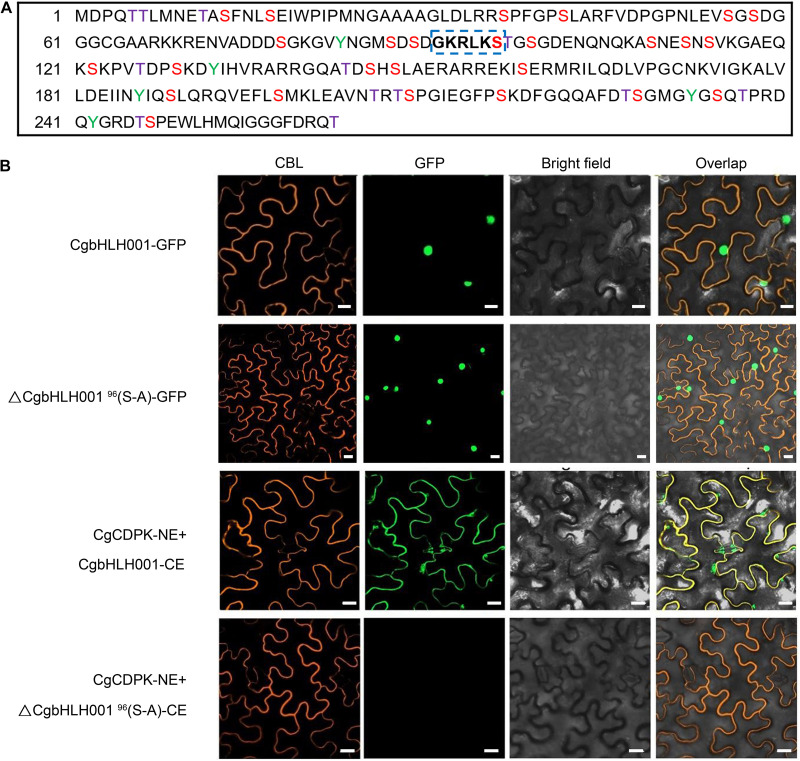
Effect of site-directed mutagenesis of CgbHLH001 on the localization and interaction with CgCDPK. **(A)** Prediction of the phosphorylation sites of CgbHLH001. The colored capital letters represent the probable phosphorylation site, purple T: threonine; red S: serine; green Y: tyrosine; the bold letters in blue dotted box represent the most probable phosphorylation site recognized by CgCDPK. **(B)** BiFC assay. ΔCgbHLH001^96^(S-A): site-directed mutation of CgbHLH001 on 96th serine to alanine. CgCDPK-NE: CgCDPK fused to the N terminus of GFP; CgbHLH001-CE, ΔCgbHLH001^96^(S-A)-CE: CgbHLH001, ΔCgbHLH001^96^(S-A) fused to the C terminus of GFP. CBL: calcineurin B-like protein, membrane marker; GFP: green fluorescent protein; bright field: visible light; overlap: merged bright field with CBL and GFP. Bar = 20 μm.

Based on the result that the kinase CgCDPK could phosphorylate CgbHLH001 *in vitro*, we predicted the possible phosphorylation sites in CgbHLH001 and found 39 potential sites (Figure 5A), among these, ^91^GKRLKS^96^ located in the N terminus is the most probable phosphorylation site by CgCDPK (Roberts and Harmon, 1992). To find out whether the change of this site would affect CgbHLH001 subcellular localization and interaction with CgCDPK, we mutated the 96th serine to alanine, and our results in BiFC assay showed that this mutation did not change the localization of CgbHLH001 in the nucleus (Figure 5B, the 2nd row). However, no fluorescence was detected any more between CgCDPK and ΔCgbHLH001^96^(S-A) in BiFC assay (Figure 5B, the 4th row). Our results suggest that ^96^Ser in the ^91^GKRLKS^96^ motif may be the potential phosphorylation site and with which the interaction between CgCDPK and CgbHLH001 may occur.

### Validation of *CgbHLH001* Function in Response to Abiotic Stress in Transgenic Tobacco

#### Phenotypic Analysis of CgbHLH001-Overexpressed Tobacco Lines

**FIGURE 6:**
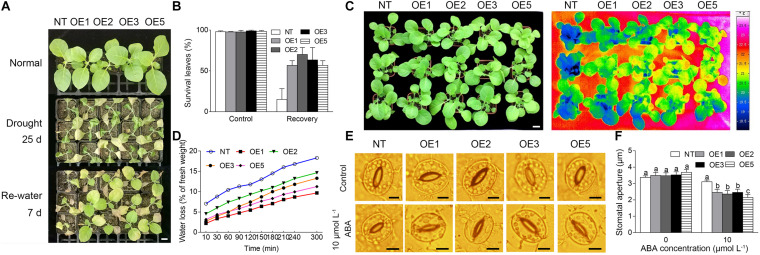
Analysis of the phenotype of transgenic tobacco lines under drought stress. **(A)** Phenotype of transgenic lines under natural drought condition. NT: non-transgenic tobacco plant; OE1, OE2, OE3, OE5: *CgbHLH001-*overexpressed transgenic lines. Normal: watering normally (1 plant for NT and each transgenic line, respectively); drought 25 days: withholding water for 25 days (3 plants for NT and each transgenic line, respectively); re-water 7 d: after re-watering for 7 days (3 plants for NT and each transgenic line, respectively). Bar = 2 cm. **(B)** Statistical analysis of survival leaves in A. Control: watering normally; recovery: after watering for 7 days. **(C)** False-color infrared image of NT plants and transgenic lines. The darker the color was, the lower the temperature was. Bar = 2 cm. **(D)** Time courses of water loss of detached leaves of NT and transgenic plants. **(E)** Effect of exogenous ABA on stomatal closure of transgenic tobacco. Bar = 5 μm. **(F)** Statistical analysis of the stomatal aperture of transgenic tobacco under ABA treatment.

To evaluate the function of *CgbHLH001* in response to stress, the transgenic tobacco lines with *CgbHLH001* overexpression were generated and identified by PCR, qRT-PCR, and seed germination (Supplementary Figures 1A–E); four T2 transgenic lines (OE1, OE2, OE3, OE5) and NT plants were subjected to drought stress. Under normal watering conditions, there was no apparently morphological difference between transgenic and NT plants; when suffered to natural drought by withholding water for 3–4 weeks, both transgenic and NT plant leaves were wilted and the NT plants were more serious than transgenic lines (Figure 6A). After being re-watered for 7 days, the NT plants were not able to recover while most plants of the four transgenic lines recovered with more than 50% of the survival green leaf percentage (Figure 6B). When 3 week-old tobacco plants were exposed to natural drought for 1 week, the leaf surface temperature of NT was lower than that of the transgenic lines and the water loss of the former was faster than that of the latter, which suggests that the transpiration of NT was stronger than that of the transgenic plants (Figures 6C,D). Meanwhile, the stomatal aperture of the transgenic leaves was significantly smaller than that of NT plants in the presence of exogenous ABA (Figures 6E,F). These data suggest that overexpression of *CgbHLH001* can improve the drought tolerance of transgenic tobacco.

#### The Physiological Performance of Transgenic Tobacco in Response to Abiotic Stress

**FIGURE 7:**
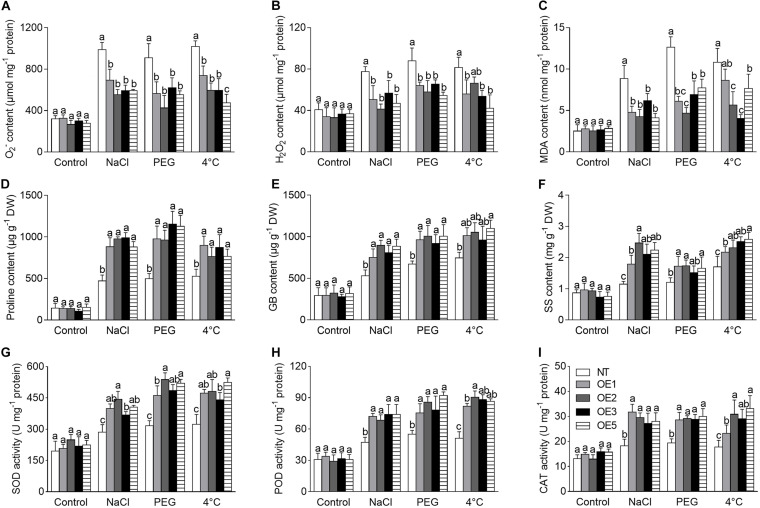
Analysis of physiological performance of transgenic tobacco lines under NaCl, PEG, and 4°C treatments. **(A–C)** ROS level and MDA content. **(D–F)** Content of osmoprotectants. **(G–I)** The activity of antioxidant enzymes. NT: non-transgenic tobacco plant; OE1, OE2, OE3, OE5: T2 transgenic tobacco line 1, 2, 3, 5. NaCl: 200 mmol⋅L^–1^ NaCl; PEG: 20% PEG 6,000. DW: dry weight. O_2_^–^: oxygen free radical; H_2_O_2_: hydrogen peroxide; MDA: malondialdehyde; GB: glycinebetaine; SS: soluble sugar; SOD: superoxide dismutase; POD: peroxidase; CAT: catalase. Different lowercase letters indicate significant difference (*P* < 0.05) between transgenic lines and NT plants within the same treatment. Values are means ± SE of three replicates.

ROS accumulation is an indicator for judgment of stress tolerance. In the present study, the ROS (including O_2_^–^ and H_2_O_2_) level was analyzed under the NaCl, PEG, and 4°C treatments, which presented a significantly higher increase in NT plants compared to that in transgenic lines (Figures 7A,B). Corresponding to this, a stronger staining by NBT (for O_2_^–^) or DAB (for H_2_O_2_) was visualized in NT plants (Supplementary Figure 2A); meanwhile, transgenic plants showed less MDA concentration than that of NT (Figure 7C), suggesting that NT plants suffered more severe damage of the cell membrane.

In consistence with the above performance, the contents of proline, GB, and SS were significantly increased in transgenic lines compared to that of NT plants under NaCl, PEG and 4°C treatments (Figures 7D–F), it implies that the osmoprotectants make contribution to the stress tolerance.

Furthermore, the activity of antioxidant enzymes, including SOD, POD, CAT, APX, and GR, which play an important role in ROS scavenging, was significantly increased in transgenic lines compared to NT plants under NaCl, PEG, or 4°C stress (Figures 7G–I and Supplementary Figures 2B,C). Meanwhile, the contents of non-enzymatic antioxidant GSH and AsA were also increased significantly in transgenic lines compared to that of NT plants under different stresses (Supplementary Figures 2D,E). Our results suggest that overexpression of *CgbHLH001* may reduce the levels of O_2_^–^, H_2_O_2_, and MDA and alleviate cell damage by increasing the activity of antioxidant enzymes and the content of antioxidants.

#### Changes of Stress-Related Genes in Transgenic Tobacco

**FIGURE 8:**
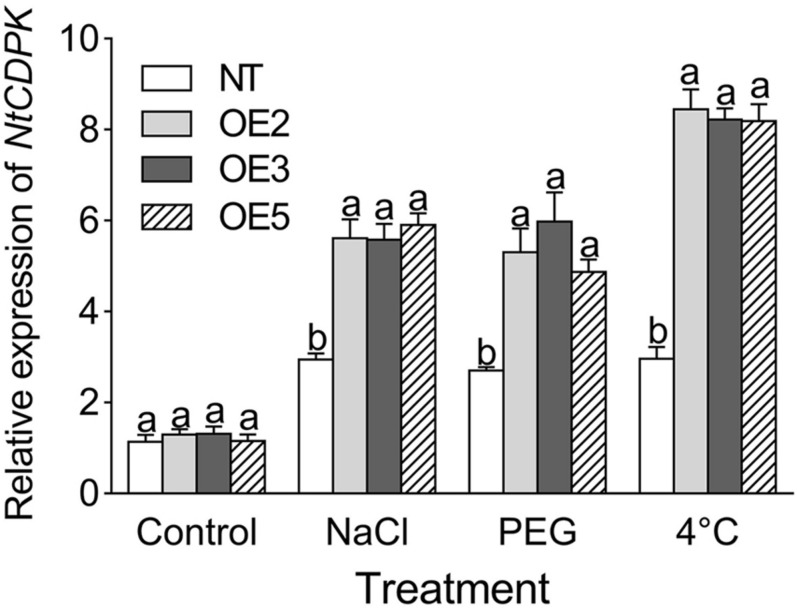
qRT-PCR analysis of the transcriptional expression pattern of *NtCDPK* in *CgbHLH001*-overexpressed tobacco. *NtCDPK*: homolog of *CgCDPK* in tobacco. NT: non-transgenic tobacco plant; OE2, OE3, OE5: *CgbHLH001*-overexpressed T2 transgenic line 2, 3, 5; control: non-stressful condition; NaCl, PEG: treatment under 300 mmol⋅L^–1^ NaCl, 15% PEG 6,000. Different lowercase letters indicate significant difference (*P* < 0.05) existing between the transgenic lines and the NT plant within the same treatment. Values are means ± SE of three biological replicates with two technical replicates of each.

To gain insight into the effect of *CgbHLH001* overexpression on the downstream genes, the transcriptional expression patterns of stress-related genes were analyzed. qRT-PCR analysis showed that the transcripts of *NtCDPK* (the homolog of *CgCDPK* in tobacco) were significantly accumulated in transgenic lines under NaCl, PEG, and 4°C treatments (Figure 8).

**FIGURE 9:**
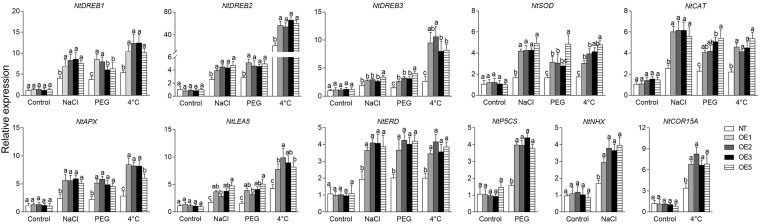
qRT-PCR analysis of the expression patterns of stress-related genes under different stresses in transgenic tobacco lines. NaCl, PEG: treatment under 200 mmol⋅L^–1^ NaCl, 20% PEG 6,000. NT: non-transgenic tobacco plant; OE1, OE2, OE3, OE5: *CgbHLH001*-overexpressed T2 transgenic line 1, 2, 3, 5. *DREB1*, *DREB2*, *DREB3*: dehydration response element-binding factor 1, 2, 3; *SOD*: superoxide dismutase; *CAT*: catalase; *APX*: ascorbate peroxidase; *LEA5*: late embryogenesis abundant protein 5; *ERD*: dehydrin of early response to dehydration; *P5CS*: delta-pyrroline-5-carboxylate synthase; *NHX*: Na^+^/H^+^ antiporter; *COR15A*: cold regulated gene 15A. All these genes are from *N. tabacum*. Different lowercase letters indicate significant difference (*P* < 0.05) existing between the transgenic lines and the NT plant within the same treatment. Values are means ± SE of three biological replicates with two technical replicates of each.

Besides, other stress-related genes, including *NtDREB1*, *2*, *3* (dehydration-responsive element-binding protein 1, 2, 3), *NtSOD* (superoxide dismutase), *NtCAT* (catalase), *NtAPX* (ascorbate peroxidase), *NtLEA5* (late embryogenesis abundant protein 5), *NtERD* (dehydrin of early response to dehydration), *NtP5CS* (delta-pyrroline-5-carboxylate synthase), *NtNHX* (Na^+^/H^+^ antiporter), and *NtCOR15A* (cold regulated gene 15A), except for the latter three genes with only PEG, NaCl, or 4°C treatment, respectively, were significantly upregulated in transgenic lines compared to that in NT plants under different treatments, while no apparent difference presented under normal conditions (Figure 9). Especially, (1) *NtDREB2*, *NtDREB3*, and *NtCOR15A*, (2) *NtDREB1*, *NtAPX*, *NtERD*, and *NtP5CS*, (3) *NtSOD*, *NtCAT*, and *NtNHX* were more actively stimulated in transgenic lines by lower temperature (1), drought stress (2), salt stress (3), respectively. Our results suggest that overexpression of *CgbHLH001* in tobacco can activate the expression of the downstream relevant genes in response to stresses.

## Discussion

It has long been documented that CDPKs actively participate in plant response to various stresses (Choi et al., 2005; Xu et al., 2010; Asano et al., 2012; Li M. Y. et al., 2020). CDPK functions are magnified by the calcium signature in the extent and duration, which may have direct effects on the phosphorylation status on the downstream targets (Ludwig et al., 2003). However, limited reports are available so far in the interaction and the phosphorylation regulation between CDPK and its substrates *in vivo* (Sebastià et al., 2004; Yip Delormel and Boudsocq, 2019). In the previous study, we reported a protein kinase CgCDPK and its potential substrate CgbHLH001 (transcription factor) in an annual halophyte *Chenopodium glaucum* (Wang et al., 2017). In the present study, we further characterized the interaction between CgCDPK and CgbHLH001; our results revealed that the phosphorylation was the probable nature of CgCDPK and CgbHLH001 interaction, and the N-terminal domain and 96th serine in it were essential for the interaction. Besides, we found that the N terminus and the bHLH domain together determined the nuclear localization of CgbHLH001. Further function analyses indicate that ectopic expression of *CgbHLH001* can confer the stress tolerance to transgenic tobacco by improving the physiological performance and stress-related gene expression. Our findings should contribute to further understanding the regulation mechanism of the CDPK signal transduction pathway in response to abiotic stresses.

As a protein kinase, CDPK generally functions by interacting with and phosphorylating the specific substrates to activate them (Nakata et al., 2009; Wang et al., 2016). However, so far only a few of transcription factors have been reported to be the targets of CDPK with phosphorylation (Boudsocq and Sheen, 2013), e.g., AtCPK32 can interact with ABF4 (ABA-responsive element-binding factor 4) *in vitro* and phosphorylate at the ^110^Ser site, which is essential for the ABA-induced transactivation in Arabidopsis (Choi et al., 2005). In the present study, the kinase activity of CgCDPK on CgbHLH001 and itself was detected and confirmed *in vitro*, suggesting that the probable nature of the CgCDPK and CgbHLH001 interaction may be the phosphorylation, because when CgbHLH001 was mutated at the 96th serine (the most probable phosphorylation site), the interaction between CgCDPK and CgbHLH001 disappeared. Based on our results, we speculate that the biological significance of the phosphorylation might be involved in the activation of CgbHLH001 and consequent translocation into the nucleus. Increasing evidence has been documented that transcription factors can control gene expression by nuclear-cytoplasmic translocation (Kaffman and O’Shea, 1999). A lipid-anchored NAC transcription factor in *Medicago falcata* is observed by translocation to the nucleus under drought stress (Duan et al., 2017). Similarly, OfbHLH164 and OfbHLH191 in *Osmanthus fragrans* may transfer to the nucleus under stimulation by a certain signal or cooperation with other transcription factors (Li Y. L. et al., 2020).

The characterization of the specific substrates of CDPK has not been well documented. Our results indicate that the N terminus in combination with the bHLH domain is indispensable for the nuclear localization of CgbHLH001. The presence of NLS is important for nuclear proteins to transport into the nucleus (Pandey and Parnaik, 1991). Classic NLSs generally have either a stretch of basic amino acids or a bipartite sequence of basic amino acids (Dingwall and Laskey, 1991). NLS analysis of CgbHLH001 in the present study showed that several clusters of predicted bipartite NLS distributed between the 43rd aa and 180th aa, which should be much important for nuclear targeting; among them, two were located in the N terminus (43rd–71st aa, 67–96th aa), one was in the bHLH domain (150–180th aa) and one was extended between two domains (136–164th aa). However, neither the N terminus nor the bHLH domain only could determine the NL. It has been reported that two domains of the bipartite sequence are required and function together to confer the nuclear location (Dingwall and Laskey, 1991). Based on the NL analysis, we further revealed that the N terminus rather than other domains was essential for the interaction, which corresponded to the absence of interaction after mutation of ^96^Ser to Ala (the probable phosphorylation site) in the N terminus. Arabidopsis CPK23 can phosphorylate SLAC1 (slowly activating anion channel) on the N-terminus exclusively, corresponding to the interaction between CPK23 and SLAC1 with the N-terminus rather than the C-terminus (Geiger et al., 2010). In maize, ZmOST1 (open stomata 1) can phosphorylate ZmKS1 (a bHLH transcription factor) or ZmKS2 on the N terminus (1–129 aa) or on the N terminus (1–149 aa) and bHLH domain (150–228 aa), respectively (Rabissi et al., 2016).

The bHLH TF has been reported to play important roles in plant growth/development, metabolism, and response to stress tolerance (Abe et al., 2003; Pillitteri and Torii, 2007; Zhao et al., 2008; Liu et al., 2013; Shi et al., 2019; Song et al., 2020; Sun et al., 2020). The best-characterized member in this family is ICE1 (inducer of CBF expression 1) in Arabidopsis, which controls the *CBF* (C-repeat binding factor) expression in frozen tolerance (Chinnusamy et al., 2003). In the present study, we explored the function of the TF *CgbHLH001* gene in stress tolerance by overexpression in tobacco. Our results showed that transgenic plants presented better phenotypic performance under drought stress, which exhibited with a higher percentage of survival leaves, lower leaf transpiration, lower water loss, and smaller stoma aperture compared to NT. The increasing evidence suggests that the physiological disturbance under stress is due to the accumulation of ROS (Miller et al., 2010; Wu et al., 2016; Mittler, 2017). Overproduction of ROS might have an adverse effect on the growth and development of plants (Alscher et al., 1997). In the present study, we found that ROS was significantly less accumulated in transgenic lines compared to NT plants under drought and salt stresses, which may avoid severe membrane damage (with much lower MDA content), suggesting that *CgbHLH001* overexpression may confer stress tolerance to transgenic tobacco by decreasing ROS production and/or enhancing the ROS scavenging ability. Generally, ROS detoxification depends on antioxidant enzymes (Ashraf, 2009; Yang J. J. et al., 2020) and antioxidants (Foyer et al., 1997); they synergetically function to scavenge the excess ROS (Donahue et al., 1997; Stepien and Klobus, 2005). Among them, SOD is considered as “the first line of defense” in converting O_2_^–^ radicals into H_2_O_2_ and O_2_ (Willekens et al., 1997). CAT, POD, GR, and APX are also the major enzymes responsible for H_2_O_2_ scavenging (Ashraf, 2009), and the latter two function coordinately in alleviation of H_2_O_2_ in the GSH and AsA cycle (Stepien and Klobus, 2005). In the present study, we found that overexpression of *CgbHLH001* significantly enhanced the activity of SOD, CAT, POD, APX, GR, and the content of AsA, GSH, which may synergetically improve the antioxidant capacity of transgenic tobacco. Not only can proline, soluble sugars, and glycinebetaine as important organic osmolytes relieve the low cellular water potential (Flowers and Colmer, 2008; Trovato et al., 2008), the latter two can also assist ROS scavenging (Fan et al., 2012). In the present study, the organic compatible solutes were accumulated much higher in transgenic lines, which may be responsible for rescuing the water loss and assist with relieving the ROS damage under drought stress.

Gene regulation under abiotic stress is subjected to multiple transcriptional cascades (Zhu, 2002; Yamaguchi-Shinozaki and Shinozaki, 2006; Liu et al., 2020), in which transcription factors may firstly be activated by upstream protein kinase (e.g., CDPK) (Gao and He, 2013) and consequently regulate downstream target genes in response to stresses (Liu et al., 2014). In our previous study, we revealed that CgCDPK overexpression in tobacco could significantly increase the *NtbHLH* (a homolog of CgbHLH001 in *N. tabacum*) expression (Wang et al., 2017). In the present study, *CgbHLH001* (TF) overexpression in tobacco also enhanced *NtCDPK* (a homolog of CgCDPK in *N. tabacum*) expression. Based on our results, we speculate that CgbHLH001 may be the specific substrate of CgCDPK *in vivo*. As transcription factors, the bHLH family has been widely reported in response to stress by regulating downstream relevant genes (Deng et al., 2017; Sun et al., 2018; Yang et al., 2019). In the present study, *CgbHLH001* overexpression significantly stimulated the transcript accumulation of various stress-related genes in transgenic tobacco under salt, drought, and cold stresses, including genes related to drought resistance (*DREB1*, *DREB2*, *DREB3*, *ERD*, *P5CS*), antioxidant enzymes (*SOD*, *CAT*, *APX*), cold resistance (*COR15A*), and salt resistance (*NtNHX*); all these genes may be regulated as the downstream targets of *CgbHLH001*. Among these tested genes, several (i.e., *NtDREB1*, *NtDREB2*, *NtDREB3*, *NtCOR15A*) actively responded to the cold stress, especially *NtDREB2* and *NtDREB3*. In *Fagopyrum tataricum*, *FtbHLH2* can also improve the cold tolerance in transgenic Arabidopsis by stimulating gene expression such as *CBF1*, *CBF3*, *COR15A*, and *RD29A* (Yao et al., 2018). It has been reported that DREB (DRE-binding protein) transcription factors can specifically bind to DRE/CRT (dehydration responsive element/C-repeat) element and be involved in both dehydration and cold responses (Shinozaki and Yamaguchi-Shinozaki, 2000; Wang et al., 2008). Overexpression of *DREB* dramatically enhances cold tolerance in transgenic plants (Miura et al., 2007; Kang et al., 2013). *RmICE1* overexpression in *Rosa multiflora* confers cold tolerance via modulating ROS levels and activating the expression of stress-responsive genes, of which *NtDREB1*, *NtDREB2*, and *NtDREB3* present higher transcription levels in the transgenic lines after 4°C treatment (Luo et al., 2020).

## Conclusion

In the present study, based on the previous identification of the interaction between CgCDPK (kinase) and CgbHLH001 (transcription factor) in halophyte *C. glaucum* (Wang et al., 2017), we further revealed that the nature of the interaction was probably the phosphorylation of CgbHLH001, which acted as the substrate of CgCDPK, and the N terminus was essential for the interaction; specifically, the ^96^Ser in motif of ^91^GKRLKS^96^ in the N terminus was crucial for the phosphorylation and interaction. Upon these, we validated the function of *CgbHLH001* in response to stress and confirmed that *CgbHLH001* overexpression could confer stress tolerance to transgenic tobacco by improving the physiological performance in scavenging excess ROS and accumulating the transcripts of stress-related genes. Our data suggest that transcription factor CgbHLH001 may be regulated by posttranslational process (phosphorylation by a protein kinase such as CDPK) and functions as a positive regulator in controlling the downstream relevant genes and improving the physiological performance in stress tolerance. Our findings would contribute to understanding the mechanism of CDPK signaling pathway in response to stress and providing candidate genes for crop improvement.

## Data Availability Statement

The datasets presented in this study can be found in online repositories. The names of the repository/repositories and accession number(s) can be found in the article/Supplementary Material.

## Author Contributions

HL and QY designed the experiment and methodology. ZZ and JW carried out most experimental work. SZ participated the partial work. HL, ZZ, and JW wrote the manuscript. All authors contributed to experimental design and data analysis, commented on the manuscript, and gave final approval for publication.

## Conflict of Interest

The authors declare that the research was conducted in the absence of any commercial or financial relationships that could be construed as a potential conflict of interest.

## Footnotes

1 http://nls-mapper.iab.keio.ac.jp/cgi-bin/NLS_Mapper_form.cgi#opennewwindow

2 http://kinasephos2.mbc.nctu.edu.tw/pu-blication.html

## Supplementary Material

Supplementary Figure 1 Generation and identification of the *CgbHLH001-*overexpressed tobacco. **(A)** Construct of *CgbHLH001*-overexpressed plant expression vector. **(B)** PCR analysis of transgenic tobacco. NT: non-transgenic tobacco plant; OE1, OE2, OE3, OE5: *CgbHLH001-*overexpressed transgenic lines; −: negative control; + : positive control; M: DL2000 DNA Marker. **(C)** RT-PCR analysis of transgenic tobacco. **(D)** qRT-PCR analysis of transgenic tobacco. **(E)** Seed germination of transgenic lines. Different lowercase letters indicate significant difference (*P* < 0.05) existing among different concentrations of the NT plant or the same transgenic line; ^∗^, ^∗∗^, ^∗∗∗^: indicate significant difference (*P* < 0.05, 0.01, 0.001) existing between NT plant and different transgenic lines within the same concentration. Values are means ± SE of four replicates with 30 seeds of each.

Supplementary Figure 2 Analysis of physiological performance of transgenic tobacco lines under NaCl, PEG and 4°C treatments. **(A)** Histochemical staining by NBT or DAB to determine the accumulation of O_2_^–^ or H_2_O_2_. Bar = 0.5 cm; **(B,C)** The activity of antioxidant enzymes; **(D,E)** The content of antioxidants. NT: non-transgenic tobacco plant; OE1, OE2, OE3, OE5: T2 transgenic tobacco line 1, 2, 3, 5. NaCl: 200 mmol⋅L^–1^ NaCl; PEG: 20% PEG 6000. GR: glutathione reductase; APX: ascorbate peroxidase; GSH: glutathione; AsA: ascorbic acid. Different lowercase letters indicate significant difference (*P* < 0.05) between transgenic lines and the NT plants within the same treatment. Values are means ± SE of three replicates.

Supplementary Table 1 Primers used in the present study.

## References

[B1] Abe H. Urao T. Ito T. Seki M. Shinozaki K. Yamaguchi-Shinozaki K. (2003). Arabidopsis AtMYC2 (bHLH) and AtMYB2 (MYB) function as transcriptional activators in abscisic acid signaling. *Plant Cell* 15 63–78. 10.1105/tpc.006130 12509522 PMC143451

[B2] Alscher R. G. Donahue J. Cramer C. L. (1997). Reactive oxygen species and antioxidant: relationships in green cells. *Physiol. Plant.* 100 224–233. 10.1111/j.1399-3054.1997.tb04778.x

[B3] Asano T. Hayashi N. Kobayashi M. Aoki N. Miyao A. Mitsuhara I. (2012). A rice calcium-dependent protein kinase OsCPK12 oppositely modulates salt-stress tolerance and blast disease resistance. *Plant J.* 69 26–36. 10.1111/j.1365-313x.2011.04766.x 21883553

[B4] Ashraf M. (2009). Biotechnological approach of improving plant salt tolerance using antioxidants as markers. *Biotechnol. Adv.* 271 84–93. 10.1016/j.biotechadv.2008.09.003 18950697

[B5] Atchley W. R. Fitch W. M. (1997). A natural classification of the basic helix-loop-helix class of transcription factors. *Proc. Natl. Acad. Sci. U S A.* 94 5172–5176. 10.1073/pnas.94.10.5172 9144210 PMC24651

[B6] Atchley W. R. Terhalle W. Dress A. (1999). Positional dependence, cliques, and predictive motifs in the bHLH protein domain. *J. Mol. Evol.* 48 501–516. 10.1007/PL00006494 10198117

[B7] Boudsocq M. Sheen J. (2013). CDPKs in immune and stress signaling. *Trends Plant Sci.* 18 30–40. 10.1016/j.tplants.2012.08.008 22974587 PMC3534830

[B8] Carretero-Paulet L. Galstyan A. Roig-Villanova I. Martínez-García J. F. Bilbao-Castro J. R. Robertson D. L. (2010). Genome-wide classification and evolutionary analysis of the bHLH family of transcription factors in Arabidopsis, poplar, rice, moss, and algae. *Plant Physiol.* 153 1398–1412. 10.1104/pp.110.153593 20472752 PMC2899937

[B9] Castilhos G. Lazzarotto F. Spagnolo-Fonini L. Bodanese-Zanettini M. H. Margis-Pinheiro M. (2014). Possible roles of basic helix-loop-helix transcription factors in adaptation to drought. *Plant Sci.* 223 1–7. 10.1016/j.plantsci.2014.02.010 24767109

[B10] Chinnusamy V. Ohta M. Kanrar S. Lee B. Hong X. H. Agarwal M. (2003). ICE1: a regulator of cold-induced transcriptome and freezing tolerance in Arabidopsis. *Gene. Dev.* 17 1043–1054. 10.1101/gad.1077503 12672693 PMC196034

[B11] Choi H. I. Park H. J. Park J. H. Kim S. Im M. Y. Seo H. H. (2005). Arabidopsis calcium-dependent protein kinase AtCPK32 interacts with ABF4, a transcriptional regulator of abscisic acid-responsive gene expression, and modulates its activity. *Plant Physiol.* 139 1750–1761. 10.1104/pp.105.069757 16299177 PMC1310556

[B12] Deng C. Y. Ye H. Y. Fan M. Pu T. L. Yan J. B. (2017). The rice transcription factors *OsICE* confer enhanced cold tolerance in transgenic Arabidopsis. *Plant Signal. Behav.* 12:e1316442. 10.1080/15592324.2017.1316442 28414264 PMC5501220

[B13] Dingwall C. Laskey R. A. (1991). Nuclear targeting sequences-a consensus? *Trends Biochem. Sci.* 16 478–481. 10.1016/0968-0004(91)90184-W 1664152

[B14] Donahue J. L. Okpodu C. M. Cramer C. L. Grabau E. A. Alscher R. G. (1997). Responses of antioxidants to paraquat in pea leaves (relationships to resistance). *Plant Physiol.* 113 249–257. 10.1104/pp.113.1.249 12223604 PMC158137

[B15] Duan M. Zhang R. X. Zhu F. G. Zhang Z. Q. Gou L. M. Wen J. Q. (2017). A lipid-anchored NAC transcription factor is translocated into the nucleus and activates *Glyoxalase I* expression during drought stress. *Plant Cell* 29 1748–1772. 10.1105/tpc.17.00044 28684428 PMC5559744

[B16] Fan W. J. Zhang M. Zhang H. X. Zhang P. (2012). Improved tolerance to various abiotic stresses in transgenic sweet potato (*Ipomoea batatas*) expressing spinach betaine aldehyde dehydrogenase. *PLoS One* 7:e37344. 10.1371/journal.pone.0037344 22615986 PMC3353933

[B17] Feller A. MacHemer K. Braun E. L. Grotewold E. (2011). Evolutionary and comparative analysis of MYB and bHLH plant transcription factors. *Plant J.* 66 94–116. 10.1111/j.1365-313X.2010.04459.x 21443626

[B18] Feng X. M. Zhao Q. Zhao L. L. Qiao Y. Xie X. B. Li H. F. (2012). The cold-induced basic helix-loop-helix transcription factor gene *MdCIbHLH1* encodes an ICE-like protein in apple. *BMC Plant Biol.* 12:22. 10.1186/1471-2229-12-22 22336381 PMC3352023

[B19] Flowers T. J. Colmer T. D. (2008). Salinity tolerance in halophytes. *N. Phyto.* 179 945–963. 10.1111/j.1469-8137.2008.02531.x 18565144

[B20] Foyer C. H. Lopez-Delgado H. Dat J. F. Scott I. M. (1997). Hydrogen peroxide- and glutathione -associated mechanisms of acclimatory stress tolerance and signaling. *Physiol. Plant.* 100 241–254. 10.1111/j.1399-3054.1997.tb04780.x

[B21] Gao X. Q. He P. (2013). Nuclear dynamics of Arabidopsis calcium-dependent protein kinases in effector-triggered immunity. *Plant Signal. Behav.* 8:e23868. 10.4161/psb.23868 23425856 PMC3956488

[B22] Geiger D. Scherzer S. Mumm P. Marten I. Ache P. Matschi S. (2010). Guard cell anion channel SLAC1 is regulated by CDPK protein kinases with distinct Ca^2+^ affinities. *Proc. Natl. Acad. Sci.* 107 8023–8028. 10.1073/pnas.0912030107 20385816 PMC2867891

[B23] Harmon A. C. Yoo B. Mccaffery C. (1994). Pseudo-substrate inhibition of CDPK, a protein kinase with a calmodulin-like domain. *Biochemistry* 33 7278–7287. 10.1021/bi00189a032 8003491

[B24] Harper J. F. Breton G. Harmon A. (2004). Decoding Ca^2+^ signals through plant protein kinases. *Annu. Rev. Plant Biol.* 55 263–288. 10.1146/annurev.arplant.55.031903.141627 15377221

[B25] Harper J. F. Huang J. F. Lloyd S. J. (1994). Genetic identification of an autoinhibitor in CDPK, a protein kinase with a calmodulin-like domain. *Biochemistry* 33 7267–7277. 10.1021/bi00189a031 8003490

[B26] Horsch R. B. Fry J. E. Hoffmann N. L. Eichholtz D. Rogers S. G. Fraley R. T. (1985). A simple and general method for transferring genes into plants. *Science* 227 1229–1231. 10.1126/science.227.4691.1229 17757866

[B27] Huang X. S. Wang W. Zhang Q. Liu J. H. (2013). A basic helix-loop-helix transcription factor, PtrbHLH, of *Poncirus trifoliata* confers cold tolerance and modulates peroxidase-mediated scavenging of hydrogen peroxide. *Plant Physiol.* 162 1178–1194. 10.1104/pp.112.210740 23624854 PMC3668048

[B28] Igarashi D. Ishida S. Fukazawa J. Takahashi Y. (2001). 14-3-3 proteins regulate intracellular localization of the bZIP transcriptional activator RSG. *Plant Cell* 13 2483–2497. 10.1105/tpc.13.11.2483 11701883 PMC139466

[B29] Institute of Botany, Academia Sinica (1983). *Iconographia Cormophytorum Sinicorum (Supplementum I).* Beijing: Science Press.

[B30] Ishida S. Yuasa T. Nakata M. Takahashi Y. (2008). A tobacco calcium-dependent protein kinase, CDPK1, regulates the transcription factor REPRESSION OF SHOOT GROWTH in response to gibberellins. *Plant Cell* 20 3273–3288. 10.1105/tpc.107.057489 19106376 PMC2630431

[B31] Ito T. Nakata M. Fukazawa J. Ishida S. Takahashi Y. (2010). Alteration of substrate specificity: the variable N-terminal domain of tobacco Ca^2+^-dependent protein kinase is important for substrate recognition. *Plant Cell* 22 1592–1604. 10.1105/tpc.109.073577 20442373 PMC2899867

[B32] Jin C. Huang X. S. Li K. Q. Yin H. Li L. T. Yao Z. H. (2016). Overexpression of a bHLH1 transcription factor of *Pyrus ussuriensis* confers enhanced cold tolerance and increases expression of stress-responsive genes. *Front. Plant Sci.* 7:441. 10.3389/fpls.2016.00441 27092159 PMC4820633

[B33] Kaffman A. O’Shea E. K. (1999). Regulation of nuclear localization: a key to a door. *Annu. Rev. Cell Dev. Biol.* 15 291–339. 10.1146/annurev.cellbio.15.1.291 10611964

[B34] Kang J. Q. Zhang H. T. Sun T. S. Shi Y. H. Wang J. Q. Zhang B. C. (2013). Natural variation of C-repeat-binding factor (CBFs) genes is a major cause of divergence in freezing tolerance among a group of *Arabidopsis thaliana* populations along the Yangtze River in China. *N. Phytol.* 199 1069–1080. 10.1111/nph.12335 23721132

[B35] Kumar K. Sinha A. K. (2013). Overexpression of constitutively active mitogen activated protein kinase kinase 6 enhances tolerance to salt stress in rice. *Rice* 6:25. 10.1186/1939-8433-6-25 24280045 PMC4883705

[B36] Kurbidaeva A. Ezhova T. Novokreshchenova M. (2014). Arabidopsis thaliana *ICE2* gene: phylogeny, structural evolution and functional diversification from *ICE1*. *Plant Sci.* 229 10–22. 10.1016/j.plantsci.2014.08.011 25443829

[B37] Li M. Y. Hu W. Ren L. C. Jia C. H. Liu J. H. Miao H. X. (2020). Identification, expression, and interaction network analyses of the *CDPK* gene family reveal their involvement in the development, ripening, and abiotic stress response in Banana. *Biochem. Genet.* 58 40–62. 10.1007/s10528-019-09916-2 31144068

[B38] Li Y. L. Li L. Ding W. J. Li H. Y. Shi T. T. Yang X. L. (2020). Genome-wide identification of *Osmanthus fragrans* bHLH transcription factors and their expression analysis in response to abiotic stress. *Environ. Exp. Bot.* 172:103990 10.1016/j.envexpbot.2020.103990

[B39] Liu W. W. Tai H. H. Li S. S. Gao W. Zhao M. Xie C. X. (2014). *bHLH122* is important for drought and osmotic stress resistance in Arabidopsis and in the repression of ABA catabolism. *N. Phytol.* 201 1192–1204. 10.1111/nph.12607 24261563

[B40] Liu W. X. Zhang F. C. Zhang W. Z. Song L. F. Wu W. H. Chen Y. F. (2013). Arabidopsis Di19 functions as a transcription factor and modulates *PR1*, *PR2*, and *PR5* expression in response to drought stress. *Mol. Plant* 6 1487–1502. 10.1093/mp/sst031 23404561

[B41] Liu Y. D. Shi Y. Zhu N. Zhong S. L. Bouzayen M. Li Z. G. (2020). SlGRAS4 mediates a novel regulatory pathway promoting chilling tolerance in tomato. *Plant Biotechnol. J.* 18 1620–1633. 10.1111/pbi.13328 31916348 PMC7292549

[B42] Lokhande V. H. Nikam T. D. Penna S. (2010). Biochemical, physiological and growth changes in response to salinity in callus cultures of *Sesuvium portulacastrum* L. *Plant Cell Tiss. Org.* 102 17–25. 10.1007/s11240-010-9699-3

[B43] Ludwig A. A. Romeis T. Jones J. D. G. (2003). CDPK-mediated signalling pathways: specificity and cross-talk. *J. Exp. Bot.* 395 181–188. 10.1093/jxb/erh008 14623901

[B44] Luo P. Li Z. Y. Chen W. Xing W. Yang J. Cui Y. Y. (2020). Overexpression of *RmICE1*, a bHLH transcription factor from *Rosa multiflora*, enhances cold tolerance via modulating ROS levels and activating the expression of stress-responsive genes. *Environ. Exp. Bot.* 178:104160 10.1016/j.envexpbot.2020.104160

[B45] Mehlmer N. Wurzinger B. Stael S. Hofmann-Rodrigues D. Csaszar E. Pfister B. (2010). The Ca^2+^-dependent protein kinase CPK3 is required for MAPK-independent salt-stress acclimation in Arabidopsis. *Plant J.* 63 484–498. 10.1111/j.1365-313X.2010.04257.x 20497378 PMC2988408

[B46] Miller G. Suzuki N. Ciftci-Yilmaz S. Mittler R. (2010). Reactive oxygen species homeostasis and signalling during drought and salinity stresses. *Plant Cell Environ.* 33 453–467. 10.1111/j.1365-3040.2009.02041.x 19712065

[B47] Mittler R. (2017). ROS are good. *Trends Plant Sci.* 22 11–19. 10.1016/j.tplants.2016.08.002 27666517

[B48] Miura K. Jin J. B. Lee J. Yoo C. Y. Stirm V. Miura T. (2007). SIZ1-mediated sumoylation of ICE1 controls CBF3/DREB1A expression and freezing tolerance in Arabidopsis. *Plant Cell* 19 1403–1414. 10.1105/tpc.106.048397 17416732 PMC1913760

[B49] Nair S. K. Burley S. K. (2000). Recognizing DNA in the library. *Nature* 404 715–717. 10.1038/35008182 10783871

[B50] Nakata M. Yuasa T. Takahashi Y. Ishida S. (2009). CDPK1, a calcium-dependent protein kinase, regulates transcriptional activator RSG in response to gibberellins. *Plant Signal Behav.* 4 372–374. 10.4161/psb.4.5.8229 19816103 PMC2676745

[B51] Ormancey M. Thuleau P. Mazars C. Cotelle V. (2017). CDPKs and 14-3-3 proteins: emerging duo in signaling. *Trends Plant Sci.* 22 263–272. 10.1016/j.tplants.2016.11.007 28065409

[B52] Pandey S. Parnaik V. K. (1991). Identification and characterization of nuclear location signal-binding proteins in nuclear envelopes. *BBA Biomembr.* 1063 81–89. 10.1016/0005-2736(91)90356-D 2015264

[B53] Pillitteri L. J. Torii K. U. (2007). Breaking the silence: three bHLH proteins direct cell-fate decisions during stomatal development. *BioEssays* 29 861–870. 10.1002/bies.20625 17691100

[B54] Rabissi A. Vilela B. Lumbreras V. Ludevid D. Culianez-Macia F. A. Pages M. (2016). Molecular characterization of maize bHLH transcription factor (ZmKS), a new ZmOST1 kinase substrate. *Plant Sci.* 253 1–12. 10.1016/j.plantsci.2016.09.003 27968978

[B55] Ranty B. Aldon D. Cotelle V. Galaud J. P. Thuleau P. Mazars C. (2016). Calcium sensors as key hubs in plant responses to biotic and abiotic stresses. *Front. Plant Sci.* 7:327. 10.3389/fpls.2016.00327 27014336 PMC4792864

[B56] Roberts D. M. Harmon A. C. (1992). Calcium modulated proteins targets of intracellular calcium signals in higher plants. *Annu. Rev. Plant Physiol. Plant Mol. Biol.* 43 375–414. 10.1146/annurev.pp.43.060192.002111

[B57] Rodriguez Milla M. A. Uno Y. Chang I. F. Townsend J. Maher E. A. Quilici D. (2006). A novel yeast two-hybrid approach to identify CDPK substrates: characterization of the interaction between AtCPK11 and AtDi19, a nuclear zinc finger protein. *FEBS Lett.* 580 904–911. 10.1016/j.febslet.2006.01.013 16438971

[B58] Schulz P. Herde M. Romeis T. (2013). Calcium-dependent protein kinases: hubs in plant stress signaling and development. *Plant Physiol.* 163 523–530. 10.1104/pp.113.222539 24014579 PMC3793034

[B59] Sebastià C. H. Hardin S. C. Clouse S. D. Kieber J. J. Huber S. C. (2004). Identification of a new motif for CDPK phosphorylation *in vitro* that suggests ACC synthase may be a CDPK substrate. *Arch. Biochem. Biophys.* 428 81–91. 10.1016/j.abb.2004.04.025 15234272

[B60] Seo J. S. Joo J. Kim M. J. Kim Y. K. Nahm B. H. Song S. I. (2011). OsbHLH148, a basic helix-loop-helix protein, interacts with OsJAZ proteins in a jasmonate signaling pathway leading to drought tolerance in rice. *Plant J.* 65 907–921. 10.1111/j.1365-313X.2010.04477.x 21332845

[B61] Shi Q. Q. Li X. Du J. T. Li X. G. (2019). Anthocyanin synthesis and the expression patterns of bHLH transcription factor family during development of the Chinese jujube fruit (*Ziziphus jujuba* Mill.). *Forests* 10:346 10.3390/f10040346

[B62] Shi R. Chiang V. L. (2005). Facile means for quantifying microRNA expression by real-time PCR. *Biotechniques* 39 519–524. 10.2144/000112010 16235564

[B63] Shinozaki K. Yamaguchi-Shinozaki K. (2000). Molecular responses to dehydration and low temperature: differences and cross-talk between two stress signaling pathways. *Curr. Opin. Plant Biol.* 3 217–223. 10.1016/S1369-5266(00)80068-0 10837265

[B64] Sivitz A. B. Hermand V. Curie C. Vert G. (2012). *Arabidopsis* bHLH100 and bHLH101 control iron homeostasis via a FIT-independent pathway. *PLoS One* 7:e44843. 10.1371/journal.pone.0044843 22984573 PMC3439455

[B65] Song C. Shan W. Kuang J. Chen J. Lu W. (2020). The basic helix-loop-helix transcription factor MabHLH7 positively regulates cell wall-modifying-related genes during banana fruit ripening. *Postharvest Biol. Tec.* 161:111068 10.1016/j.postharvbio.2019.111068

[B66] Stepien P. Klobus G. (2005). Antioxidant defense in the leaves of C3 and C4 plants under salinity stress. *Physiol. Plant.* 125 31–40. 10.1111/j.1399-3054.2005.00534.x

[B67] Sun W. J. Jin X. Ma Z. T. Chen H. Liu M. Y. (2020). Basic helix-loop-helix (*bHLH*) gene family in Tartary buckwheat (*Fagopyrum tataricum*): Genome-wide identification, phylogeny, evolutionary expansion and expression analyses. *Int. J. Biol. Macromol.* 155 1478–1490. 10.1016/j.ijbiomac.2019.11.126 31734362

[B68] Sun X. Wang Y. Sui N. (2018). Transcriptional regulation of bHLH during plant response to stress. *Biochem. Biophys. Res. Commun.* 503 397–401. 10.1016/j.bbrc.2018.07.123 30057319

[B69] Trovato M. Mattioli R. Costantino P. (2008). Multiple roles of proline in plant stress tolerance and development. *Rend. Lincei* 19 325–346. 10.1007/s12210-008-0022-8

[B70] Ubersax J. A. Ferrell J. E. (2007). Mechanisms of specificity in protein phosphorylation. *Nat. Rev. Mol. Cell Biol.* 8 530–541. 10.1038/nrm2203 17585314

[B71] Uno Y. Rodriguez Milla M. A. Maher E. Cushman J. C. (2009). Identification of proteins that interact with catalytically active calcium dependent protein kinases from Arabidopsis. *Mol. Genet. Genomics* 281 375–390. 10.1007/s00438-008-0419-1 19130088

[B72] Wang J. Cheng G. Wang C. He Z. Z. Lan X. X. Zhang S. Y. (2017). The bHLH transcription factor CgbHLH001 is a potential interaction partner of CDPK in halophyte *Chenopodium glaucum*. *Sci. Rep.* 7:8441. 10.1038/s41598-017-06706-x 28814803 PMC5559460

[B73] Wang L. L. Yu C. C. Xu S. L. Zhu Y. G. Huang W. C. (2016). OsDi19-4 acts downstream of OsCDPK14 to positively regulate ABA response in rice. *Plant Cell Environ.* 39 2740–2753. 10.1111/pce.12829 27627618

[B74] Wang Q. Y. Guan Y. C. Wu Y. R. Chen H. L. Chen F. Chu C. C. (2008). Overexpression of a rice *OsDREB1F* gene increases salt, drought, and low temperature tolerance in both Arabidopsis and rice. *Plant Mol. Biol.* 67 589–602. 10.1007/s11103-008-9340-6 18470484

[B75] Willekens H. Chamnongpol S. Davey M. Schraudner M. Langebartels C. Montagu M. V. (1997). Catalase is a sink for H_2_O_2_ and is indispensable for stress defence in C3 plants. *EMBO J.* 16 4806–4816. 10.1093/emboj/16.16.4806 9305623 PMC1170116

[B76] Wu H. L. Chen C. L. Du J. Liu H. F. Cui Y. Zhang Y. (2012). Co-overexpression FIT with *AtbHLH38* or *AtbHLH39* in Arabidopsis-enhanced cadmium tolerance via increased cadmium sequestration in roots and improved iron homeostasis of shoots. *Plant Physiol.* 158 790–800. 10.1104/pp.111.190983 22184655 PMC3271767

[B77] Wu H. Fu B. Sun P. P. Xiao C. Liu J. H. (2016). A NAC transcription factor represses putrescine biosynthesis and affects drought tolerance. *Plant Physiol.* 172 1532–1547. 10.1104/pp.16.01096 27663409 PMC5100760

[B78] Xu J. Tian Y. S. Peng R. H. Xiong A. S. Bo Z. Jin X. F. (2010). AtCPK6, a functionally redundant and positive regulator involved in salt/drought stress tolerance in Arabidopsis. *Planta* 231 1251–1260. 10.1007/s00425-010-1122-0 20217124

[B79] Yamaguchi-Shinozaki K. Shinozaki K. (2006). Transcriptional regulatory networks in cellular responses and tolerance to dehydration and cold stresses. *Annu. Rev. Plant Biol.* 57 781–803. 10.1146/annurev.arplant.57.032905.105444 16669782

[B80] Yang J. J. Zhang G. Q. An J. Li Q. X. Chen Y. H. Zhao X. Y. (2020). Expansin gene *TaEXPA2* positively regulates drought tolerance in transgenic wheat (*Triticum aestivum* L.). *Plant Sci.* 298:110509. 10.1016/j.plantsci.2020.110596 32771153

[B81] Yang J. Zhang X. Blumenthal R. M. Cheng X. D. (2020). Detection of DNA modifications by sequence-specific transcription factors. *J. Mol. Biol.* 432 1661–1673. 10.1016/j.jmb.2019.09.013 31626807 PMC7156337

[B82] Yang X. Wang R. Hu Q. L. Li S. L. Mao X. D. Jing H. H. (2019). *DlICE1*, a stress-responsive gene from *Dimocarpus longan*, enhances cold tolerance in transgenic Arabidopsis. *Plant Physiol. Bioch.* 142 490–499. 10.1016/j.plaphy.2019.08.007 31442880

[B83] Yao P. F. Sun Z. X. Li C. L. Zhao X. R. Li M. F. Deng R. Y. (2018). Overexpression of *Fagopyrum tataricum FtbHLH2* enhances tolerance to cold stress in transgenic Arabidopsis. *Plant Physiol. Bioch.* 125 85–94. 10.1016/j.plaphy.2018.01.028 29427891

[B84] Yip Delormel T. Boudsocq M. (2019). Properties and functions of calcium-dependent protein kinases and their relatives in *Arabidopsis thaliana*. *N. Phytol.* 224 585–604. 10.1111/nph.16088 31369160

[B85] Yuan Y. X. Wu H. L. Wang N. Li J. Zhao W. N. Du J. (2008). FIT interacts with AtbHLH38 and AtbHLH39 in regulating iron uptake gene expression for iron homeostasis in Arabidopsis. *Cell Res.* 18 385–397. 10.1038/cr.2008.26 18268542

[B86] Zhai Y. Zhang L. Xia C. Fu S. Zhao G. Jia J. (2016). The wheat transcription factor, TabHLH39, improves tolerance to multiple abiotic stressors in transgenic plants. *Biochem. Biophys. Res. Commun.* 473 1321–1327. 10.1016/j.bbrc.2016.04.071 27091431

[B87] Zhao M. Morohashi K. Hatlestad G. Grotewold E. Lloyd A. (2008). The TTG1-bHLH-MYB complex controls trichome cell fate and patterning through direct targeting of regulatory loci. *Development* 135 1991–1999. 10.1242/dev.016873 18434419

[B88] Zhu J. K. (2002). Salt and drought stress signal transduction in plants. *Annu. Rev. Plant Biol.* 53 247–273. 10.1146/annurev.arplant.53.091401.143329 12221975 PMC3128348

[B89] Zou J. J. Li X. D. Ratnasekera D. Wang C. Liu W. X. Song L. F. (2015). Arabidopsis calcium dependent protein kinase8 and catalase3 function in ABA signaling and H_2_O_2_ homeostasis in stomatal guard cells under drought stress. *Plant Cell* 27 1445–1460. 10.1105/tpc.15.00144 25966761 PMC4456645

