# Supplementary material for: Investigation of the Nature of CgCDPK and CgbHLH001 Interaction and the Function of bHLH Transcription Factor in Stress Tolerance in Chenopodium glaucum
Source: Front Plant Sci. 2021 Jan 22;11:603298. doi: 10.3389/fpls.2020.603298 (PMC7862342; doi:10.3389/fpls.2020.603298)
Supplement: Supplementary Figure 1 — Generation and identification of the CgbHLH001-overexpressed tobacco. (A) Construct of CgbHLH001-overexpressed plant expression vector. (B) PCR analysis of transgenic tobacco. NT: non-transgenic tobacco plant; OE1, OE2, OE3, OE5: CgbHLH001-overexpressed transgenic lines; −: negative control; + : positive control; M: DL2000 DNA Marker. (C) RT-PCR analysis of transgenic tobacco. (D) qRT-PCR analysis of transgenic tobacco. (E) Seed germination of transgenic lines. Different lowercase letters indicate significant difference (P < 0.05) existing among different concentrations of the NT plant or the same transgenic line; ∗, ∗∗, ∗∗∗: indicate significant difference (P < 0.05, 0.01, 0.001) existing between NT plant and different transgenic lines within the same concentration. Values are means ± SE of four replicates with 30 seeds of each. [file Data_Sheet_1.docx]

Supplementary Material

**SUPPLEMENTARY TABLE 1 Primers used in the present study.**

| Gene name | Primer sequence（5’-3’） | |
| --- | --- | --- |
|  | Forward | Reverse |
| Super1300-CgbHLH001(1-146)-GFP | GCTCTAGAATGGATCCACAGACGACGCT | GGGGTACCATGGCTATCAGTTGCTTGACC |
| Super1300-CgbHLH001(1-197)-GFP | GCTCTAGAATGGATCCACAGACGACGCT | GGGGTACCAAACTCAACTTGACGCTGCAATG |
| Super1300-CgbHLH001(147-263)-GFP | GCTCTAGAAGCCTTGCTGAAAGGGCTAGA | GGGGTACCTGTTTGTCTGTCAAAACCACCA |
| Super1300-CgbHLH001(147-197)-GFP | TGCAGGGGCCCGGGGTCGACAGCCTTGCTGAAAGGGCTAGAA | ACTAGTATTTAAATGTCGACAAACTCAACTTGACGCTGCAAT |
| Super1300-CgbHLH001(198-263)-GFP | GCTCTAGACTCTCCATGAAATTAGAAGCTGTC | GGGGTACCTGTTTGTCTGTCAAAACCACCA |
| pSPY-CgbHLH001(1-146)-CE | GCGTCGACATGGATCCACAGACGACGCT | GGGGTACCATGGCTATCAGTTGCTTGACC |
| pSPY-CgbHLH001(1-197)-CE | GCGTCGACATGGATCCACAGACGACGCT | GGGGTACCAAACTCAACTTGACGCTGCAATG |
| pSPY-CgbHLH001(147-263)-CE | GCGTCGACAGCCTTGCTGAAAGGGCTAGA | GGGGTACCTGTTTGTCTGTCAAAACCACCA |
| pSPY-CgbHLH001(198-263)-CE | GCGTCGACCTCTCCATGAAATTAGAAGCTGTC | GGGGTACCTGTTTGTCTGTCAAAACCACCA |
| pPR3-N-CgbHLH001(1-146) | ATTAACAAGGCCATTACGGCCATGGATCCACAGACGACGCT | AACTGATTGGCCGAGGCGGCCATGGCTATCAGTTGCTTGACC |
| pPR3-N-CgbHLH001(1-197) | ATTAACAAGGCCATTACGGCCATGGATCCACAGACGACGCT | AACTGATTGGCCGAGGCGGCCAAACTCAACTTGACGCTGCAATG |
| pPR3-N-CgbHLH001(147-263) | ATTAACAAGGCCATTACGGCCAGCCTTGCTGAAAGGGCTAGA | AACTGATTGGCCGAGGCGGCCTCATGTTTGTCTGTCAAAACCACCAC |
| pPR3-N-CgbHLH001(198-263) | ATTAACAAGGCCATTACGGCCCTCTCCATGAAATTAGAAGCTGTC | AACTGATTGGCCGAGGCGGCCTCATGTTTGTCTGTCAAAACCACCAC |
| CgbHLH001(mutated) | GGTAAAAGGCTCAAAGCCACAGGATCCGGAGAT | ATCTCCGGATCCTGTGGCTTTGAGCCTTTTACC |
| *NtCDPK* (qRT-PCR) | ACAGAGGAGTTACGGCAAGG | CAGAGGGCCAAGGTTCACTT |
| *NtDREB1* (qRT-PCR) | CAGGTAAGTGGGTGTGTGAAGTGA | CGAGTCGGCGAAATTGAGAC |
| *NtDREB2* (qRT-PCR) | GGGTCGTTCTGCTTGCTTGA | GAGTAGTGCCTGAATCTGCTTCTTC |
| *NtDREB3* (qRT-PCR) | CAGGCAAGTGGGTTTCTGAAGTC | GAGGAAGCAGGAACAGGCAACT |
| *NtSOD* (qRT-PCR) | CTCCTACCGTCGCCAAAT | GCCCAACCAAGAGAACCC |
| *NtCAT* (qRT-PCR) | AGGTACCGCTCATTCACACC | AAGCAAGCTTTTGACCCAGA |
| *NtAPX* (qRT-PCR) | CAAATGTAAGAGGAAACTCAGAGGA | CAGCCTTGAGCCTCATGGTACCG |
| *NtLEA5* (qRT-PCR) | TTGAATCTGGGGTTTTGGTT | GGAAGCATTGACGAGCTAGG |
| *NtERD10C* (qRT-PCR) | AACGTGGAGGCTACAGATCG | GTTCCTCTTGGGCATGAGTT |
| *NtP5CS* (qRT-PCR) | GAACGGAGGTTGCTGATGGA | TCCCACTTCGGACTGCTAGA |


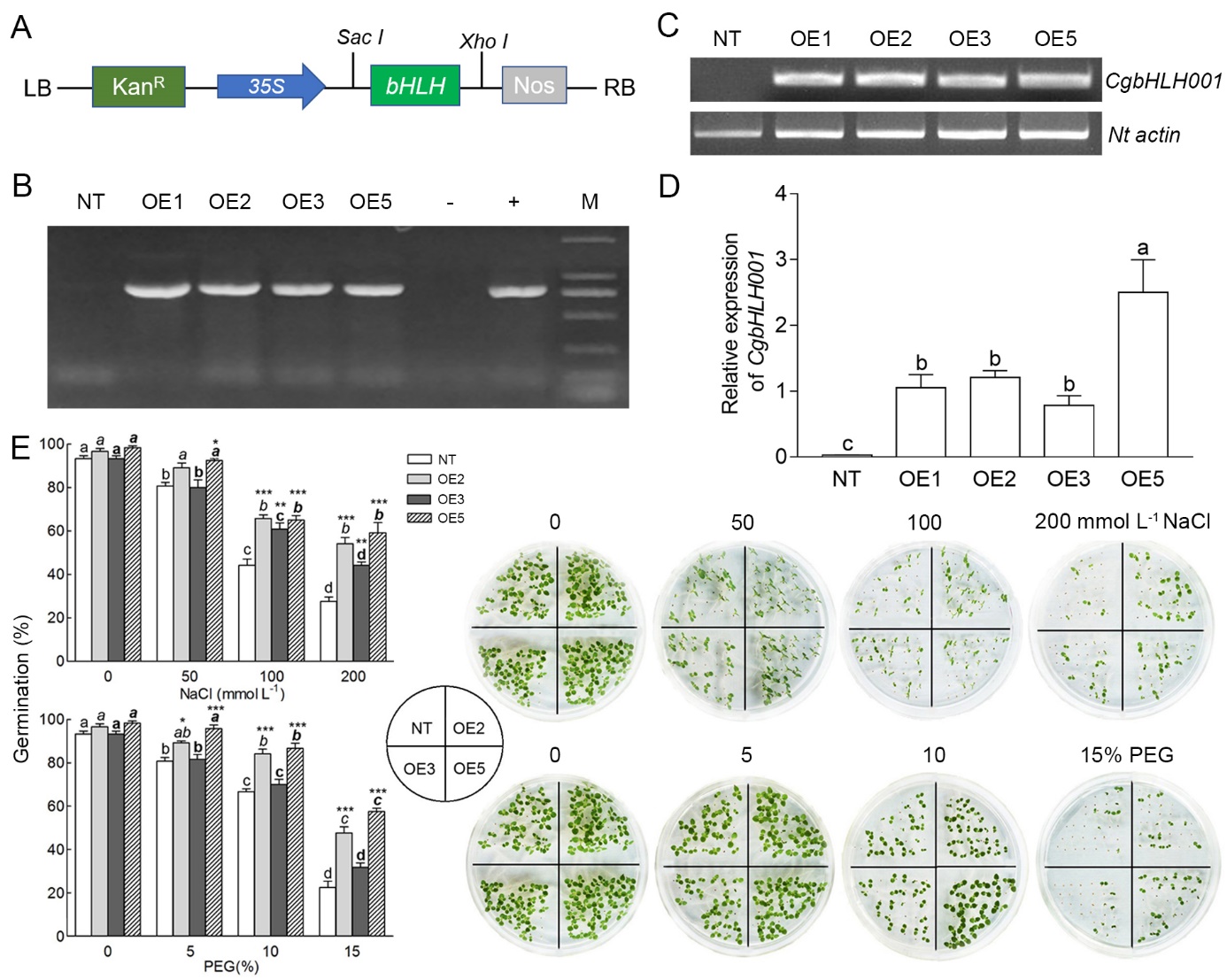


**SUPPLEMENTARY FIGURE 1 Generation and identification of the *CgbHLH001-*overexpressed tobacco.** **(A)** Construct of *CgbHLH001*-overexpressed plant expression vector. **(B)** PCR analysis of transgenic tobacco. NT: non-transgenic plant; OE1, OE2, OE3, OE5: *CgbHLH001-*overexpressed transgenic lines; -: negative control; +: positive control; M: DL2000 DNA Marker. **(C)** RT-PCR analysis of transgenic tobacco. **(D)** qRT-PCR analysis of transgenic tobacco. **(E)** Seed germination of transgenic lines. Different lowercase letters indicate significant difference (*P*<0.05) existing among different concentrations of the NT plant or the same transgenic line; *, **, ***: indicate significant difference (*P*<0.05, 0.01, 0.001) existing between NT plant and different transgenic lines within the same concentration. Values are means ± SE of four replicates with 30 seeds of each.


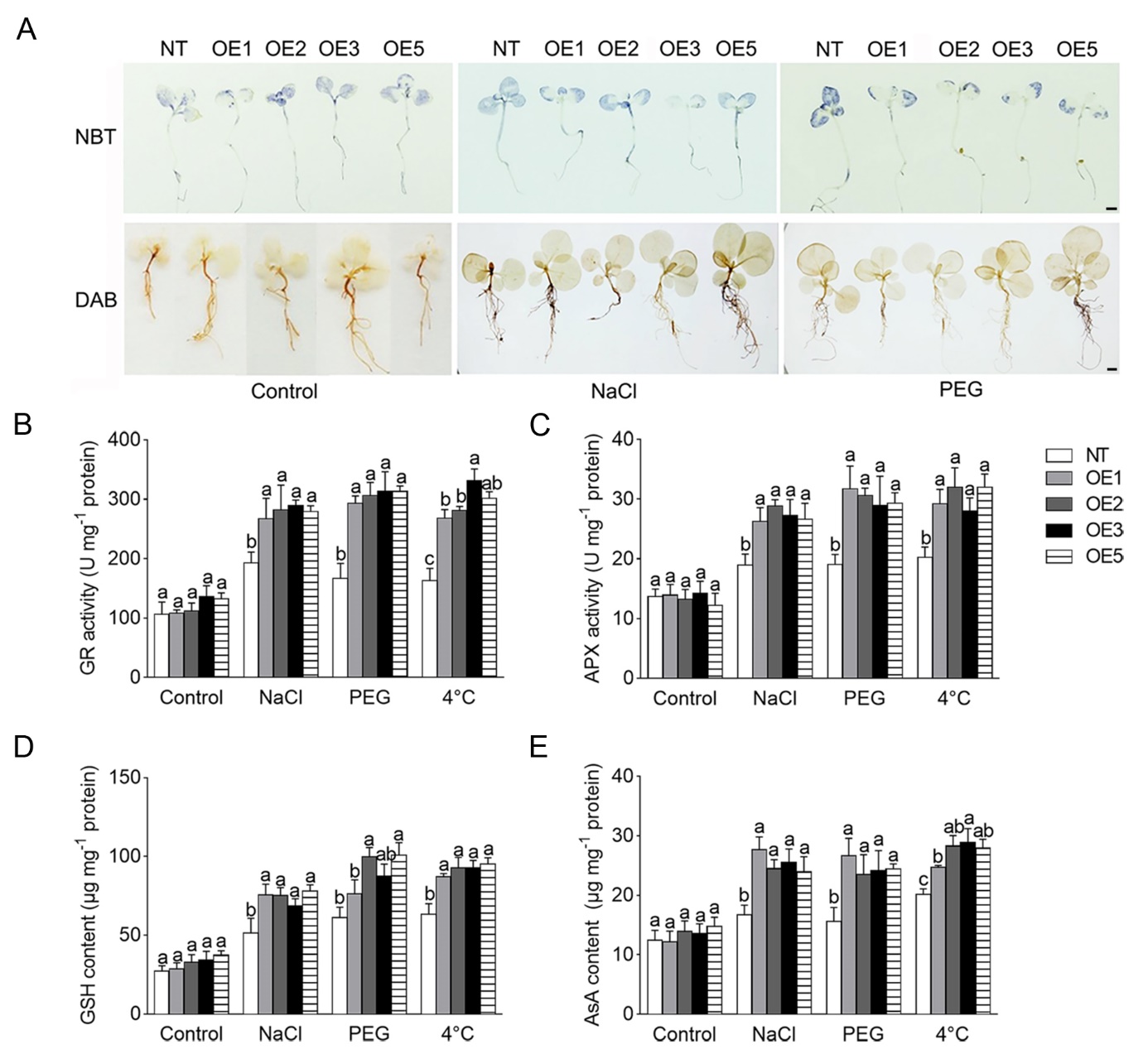


**SUPPLEMENTARY FIGURE 2 Analysis of physiological performance of transgenic tobacco lines under NaCl, PEG and 4°C treatments.** **(A)** Histochemical staining by NBT or DAB to determine the accumulation of O_2_^−^ or H_2_O_2_. Bar = 0.5 cm; **(B, C)** The activity of antioxidant enzymes; **(D, E)** The content of antioxidants. NT: non-transgenic tobacco plant; OE1, OE2, OE3, OE5: T2 transgenic tobacco line 1, 2, 3, 5. NaCl: 200 mmol·L^-1^ NaCl; PEG: 20% PEG 6000. GR: glutathione reductase; APX: ascorbate peroxidase; GSH: glutathione; AsA: ascorbic acid. Diﬀerent lowercase letters indicate significant diﬀerence (*P*<0.05) between transgenic lines and the NT plants within the same treatment. Values are means ± SE of three replicates.
